# Climate change and modernization drive structural realignments in European grain production

**DOI:** 10.1038/s41598-022-10670-6

**Published:** 2022-05-05

**Authors:** Z. Pinke, B. Decsi, A. Jámbor, M. K. Kardos, Z. Kern, Z. Kozma, T. Ács

**Affiliations:** 1 Department of Physical Geography, Eötvös Loránd University, Pázmány Péter sétány 1/C, Budapest, 1117 Hungary; 2 Department of Sanitary and Environmental Engineering, Budapest University of Technology and Economics, Műegyetem rkp. 3, Budapest, 1111 Hungary; 3 Department of Agribusiness, Corvinus University of Budapest, Fővám tér 8, Budapest, 1093 Hungary; 4 Research Centre for Astronomy and Earth Sciences, Institute for Geological and Geochemical Research, Budaörsi út 45, Budapest, 1112 Hungary

**Keywords:** Climate-change impacts, Environmental impact

## Abstract

Charting the long-term trends in European wheat and maize yields and harvested areas and the relation of yields to climatic and economic drivers, two profound spatial processes become apparent. One consequence of the relatively late modernization of Eastern Europe has been to shift the focus of grain production from West to East. The warming trend prevailing over the past decades in the summer and winter seasons has been accompanied by a South to North shift in the harvested areas. The combination of these two processes has meant that the north-eastern sector of the European grain chessboard has emerged as the main beneficiary. There, the relatively low sensitivity of cereals to climatic change plus high economic growth rates have been accompanied by the most dynamic increases in cereal yields on the continent. As a result, a modern version of the 3000 year-old grain distribution system of the Ancient World is being restored before our eyes. One noteworthy finding is that increasing January–March temperatures have had a significant positive impact on wheat yields from Northern to South-Eastern Europe, and this is, at least in part, compensating for the negative impact of summer warming.

## Introduction

Wheat (*Triticum aestivum*) provides almost 20% of humanity’s energy intake^1,2^. Providing ca. one-third of the world’s annual wheat harvest, Europe is a centre of global wheat production, while if one considers wheat exports, European countries accounted for 53% of global wheat exports between 2013 and 2017^3^. Maize (*Zea mays L.*) is the primary forage of livestock globally, and a staple food in certain tropical and sub-tropical areas^4^. As with wheat, Europe’s share of total global maize production (ca. 11%), is far exceeded by its share of global maize exports (30% between 2013 and 2017)^3^. What is more, in global terms, wheat and maize production has grown most dynamically in Eastern Europe in recent decades^5–7^. Meanwhile, the wheat production of other leading producers, e.g. Australia, China and a major part of India, and maize production in Northern China, Northern India and Central America has stagnated^3,8^. As a result, Eastern European countries have taken over the leading positions in global export rankings in recent years^3^.

Eastern Europe as a supplier of grain to other regions has a long story^9^. The fertile croplands of the Black Sea hinterland had been integrated into the Mediterranean Greek trading system by the early 2nd millennium BC. The steady, regular supply of grain from the Black Sea Basin was underway, and was to become by the latest in the fourth century BC^10,11^ a key resource maintaining the relatively high population density in the Eastern Mediterranean world. Subsequent developments notwithstanding, for example, hegemony over the majority of Black Sea region by, among others, the Roman, Byzantine, Ottoman and Russian empires, the grain of the Eastern European steppe was an integral part of Eastern Mediterranean food security until the communist takeover^12,13^. What is more, Baltic grain, from Poland, Lithuania and other Eastern entities was a key component in Scandinavian and Western European food security from the thirteenth century till the nineteenth century “overseas grain invasion”^14,15^. The biophysical basis of the high grain production potential in Eastern Europe is that some of the richest top soils in the world (Chernozems, Mollisols in the USDA taxonomy) cover vast areas in the region^16^. This outstanding natural soil fertility is not just important in and of itself, but plays a key role in climate resilience, mitigating extremities associated with the continental climate that characterizes Central and Eastern Europe^17^. However, even these relatively favorable natural conditions could not mitigate or compensate for the extreme effects of collectivization, the liquidation of earlier elites and the disengagement from the world economy prevalent under the communist regimes^18^. Due to technological disruption occasioned by this, the productivity of Eastern Europe could not evolve and acute food shortage moreover famines became commonplace in communist regimes^19,20^.

The dynamic growth in grain yields brought about by the Green Revolution of the mid-twentieth century had slowed down by the 1990s, reaching a plateau, or even turning to a decrease in key agricultural regions of the world^21^; this, in turn, has caused serious concerns about global food security^22,23^. The direct and indirect effects of climate change^24,25^, the diminution of freshwater resources^26,27^, the simplification of crop rotation and decreasing artificial fertilizer use^28^ are mentioned among the main drivers of this negative process. Droughts, diminishing freshwater supplies^27,29–31^, and soil degradation due to poor land management in industrialized agriculture^17^ are core problems over the greater part of Europe. For these reasons, tens of millions of hectares of fertile soils have suffered catastrophic erosion across Eastern Europe^17^. Therefore, adaptation to climate change, and specifically an enhanced ability to adapt to heatwaves, have now become top priorities for European agriculture^25,32,33^. Statistical and process-based model analyses suggest that a 1 °C rise in global mean temperature will lead to a 4.1 to 6.4% decrease in wheat yields if the sown area remains constant and there is no fundamental improvement in technology^34,35^. Furthermore, drylands such as those surrounding the Mediterranean Basin, or continental fields, can expect a greater degree of warming than humid lands^36,37^, and it is precisely in these drylands, rather than humid zones, where increasing temperature accounts for a significantly higher degree of interannual fluctuation in cereal yields^29,38^. Besides these negative effects, current environmental changes may have positive impacts on certain plant communities, including cultivated plants in several regions. Comparing plants utilizing different photosynthetic pathways, the general expectation is that C_3_ plants, representing ca. 90% of plant species, including wheat, have been beneficiaries of the elevated atmospheric CO_2_ concentration, while C_4_ plants, including maize, are relatively unresponsive to rising CO_2_ levels^39,40^. That said, there is a broad consensus that the expected impacts of climate change on grain production will be more positive in Northern Europe than other parts of the continent^39,41^. This includes increasing precipitation counterbalancing the negative impact of warming in the British Isles and the Atlantic coasts of Western Europe^42^. Beyond this, the lengthening of growing seasons due to warming generates a shift in crop cultivation observable along the northern border of the European croplands^43^. These regional differences in vulnerability also manifest themselves on the map of potential wheat and maize yields^44^. Taken together, the importance of Europe in global food security, and the rapid change in production structure necessitate the identification of yield trends and the main drivers behind changes in yields over longer periods on the continent^33^. Nevertheless, a detailed analysis of the overall pattern of trends in the wheat and maize yields and harvested areas of European countries over the past 25 years is still lacking.

The expected agricultural development including yield increase in cropland farming may come from the improving environmental conditions and the growth of the invested human and material resources^45^. The estimated share of human input in wheat and maize production ranged between 91–98 and 91–100%, respectively, in the EU countries in 2008^46^. To assess the measure of agricultural inputs (utilized material resources) in a uniform way, the total factor productivity (TFP), the most popular measure of agricultural productivity, was invented^47^. The TFP index is defined as the ratio of output and inputs^48^. The calculation of inputs to the agricultural TFP is based on net capital stock intensity, the number of farm labourers, the extent of the land, the amount of or total metric horse-power of farm machinery, and the sum of fertilizers and livestock^47,49^. Within the framework of a science-based agriculture^50^ and the transformation of environmental conditions, however, the “residual” part of the outputs that cannot be accounted for the inputs^48^ may increase precipitously to an extreme degree, and the listed input factors cannot bring a profound understanding of agricultural productivity growth^50,51^.

Considering these points a preliminary examination of the available TFP indices was made^49^, and it was found that there are no consistent data on agricultural productivity for all European countries over the long term (Supplementary Table S8). Self and Grabowski (2007)^52^ suggest that the “improvements in agricultural technology have a significant impact on long-run economic growth”. Moreover, a strong dependence of agriculture development on the macroeconomic environment^53–56^ and bidirectional correlations are identified between economic growth and agricultural productivity increase in many studies, suggesting causal^44,57,58^ and non-causal connections^59^. Thus, in this study, gross domestic product (GDP) per capita, as a proxy of technological and institutional background of agricultural production was employed.

In an attempt to fill in the gaps mentioned above, long-term trends in the wheat and maize yields and harvested areas of all European countries are charted for 1993–2017, and for 1961–1991, in the case of territorially continuous European countries. Following this, the associations will be mapped between (1) precipitation and temperature, and GDP change, as explanatory factors, and (2) wheat and maize yields, as response variables. Due to the fact that available time series do not differentiate between wheat sown in autumn and spring for Europe, and further considering that an overwhelming majority of European wheat is sown in autumn (> 95%)^60^, here wheat was examined as winter wheat. A hypothesis of the research is that rising summer temperatures have had an increasingly negative impact on cereal yields in the majority of Europe. Consequently, and conversely, the positive impact of precipitation may be expected to increase significantly in summer months. Finally, an intensive yield convergence driven by economic, including agrotechnological development is also predicted for the post-communist European states, with its beginnings datable to the mid-1990s.

## Results

### Global cereal production

**Figure 1:**
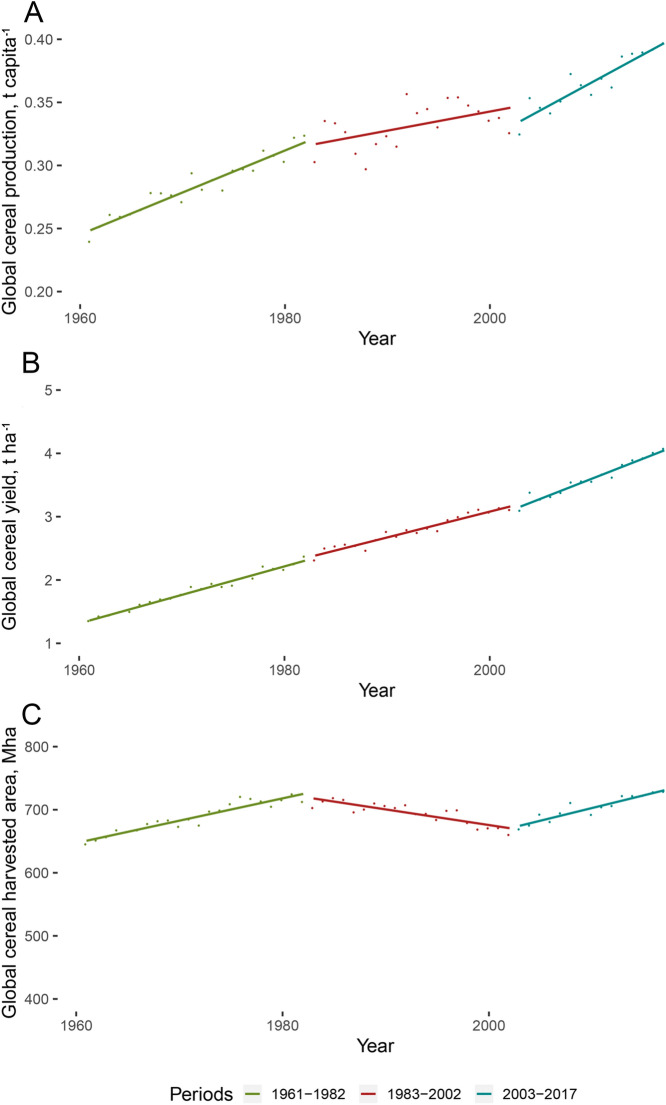
Phases in the changing rates of increase in year-to-year world cereal production per capita (**A**), global average of cereal yields (tonne per hectare) (**B**) and global area under cereal cultivation (**C**) (1961–2017). Mha = million hectares. Data source: FAOSTAT^3^ and World Bank^61^.

According to World Bank calculations on the basis of data collected by the FAO, between 1993 and 2017 global cereal production increased from an estimated 1.9 to 3.0 billion tons, a jump of 58%, while the global population rose from 5.5 to 7.5 billion, that is, a growth of 36%^61^. This spectacular growth in production was based primarily on efficiency gains (Δt ha^−1^ = 48.4%), since the area under cereal cultivation only grew by an estimated 5.0% in this period^3^. As a result, global average per capita cereal production reached an estimated 0.4 metric tonnes in 2017 (Fig. 1), that is, a level 16% higher than 25 years previously. The average year-to-year rate of increase in global cereal production per capita and global cereal yield (t ha^−1^) reached 1.4% and 2.6% between 1961 and 1982, respectively; slowing down to an average 0.2% tonnes per capita increase and 1.5% yield increase in the next 20 year period (1983–2002), then rising back to a 1.6% tonnes per capita increase in production, and 1.8% yield growth between 2003 and 2017^61^. While global cereal production almost quadrupled between 1961 and 2017, the cereal area harvested expanded by an estimated 12.9% in this 57 year period^3,61^.

### European wheat and maize production

**Figure 2:**
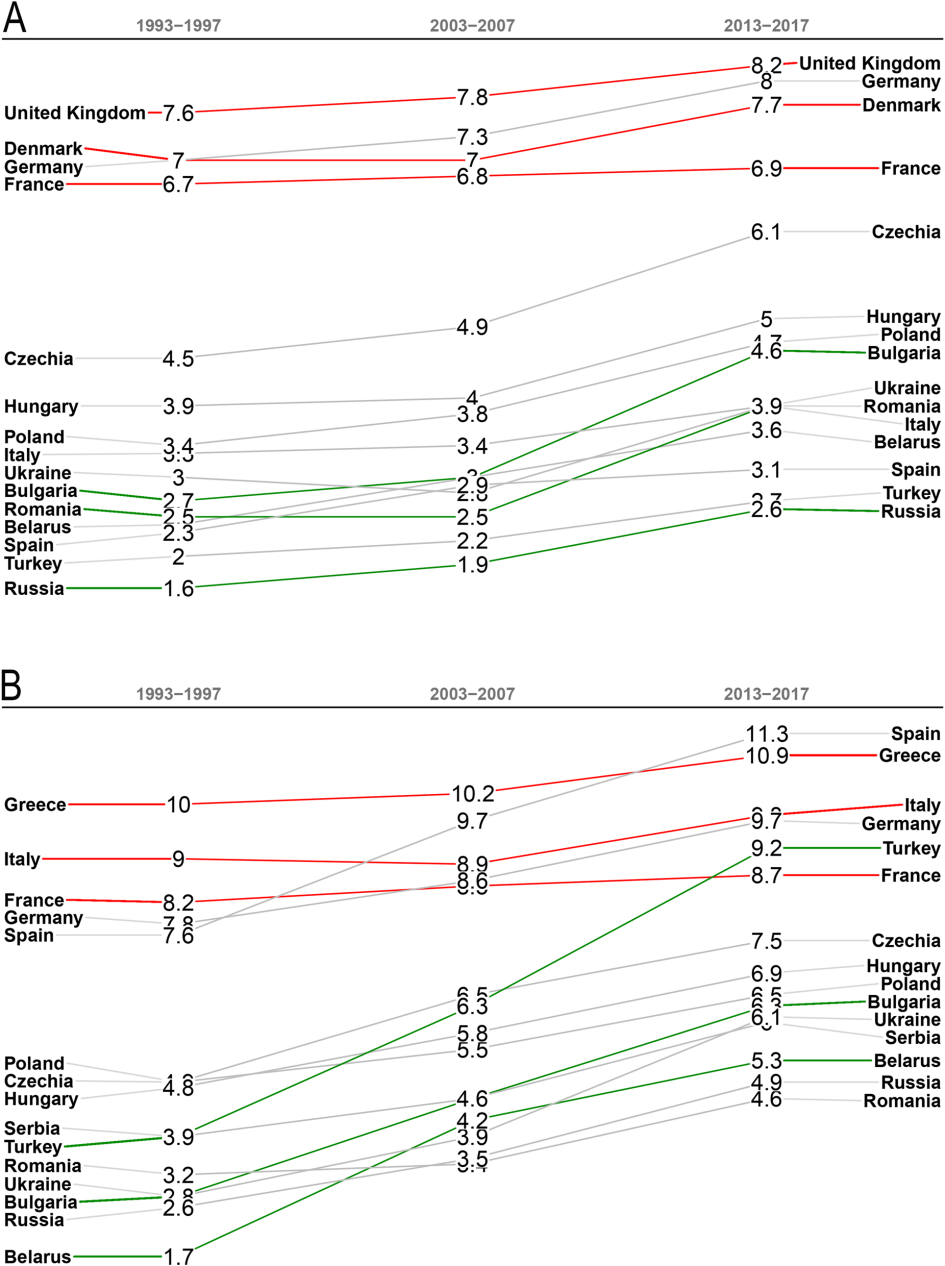
Rank table changes of (**A**) wheat and (**B**) maize yields (t ha^−1^) in the 15 biggest European producers between the periods 1993–1997 and 2013–2017. Green lines indicate the three countries with the highest yield growth and red lines the three countries with the lowest yield growth. Supplementary Figs. S1 and S2 show the total European yield rankings. Data sources: FAOSTAT^3^.

**Figure 3:**
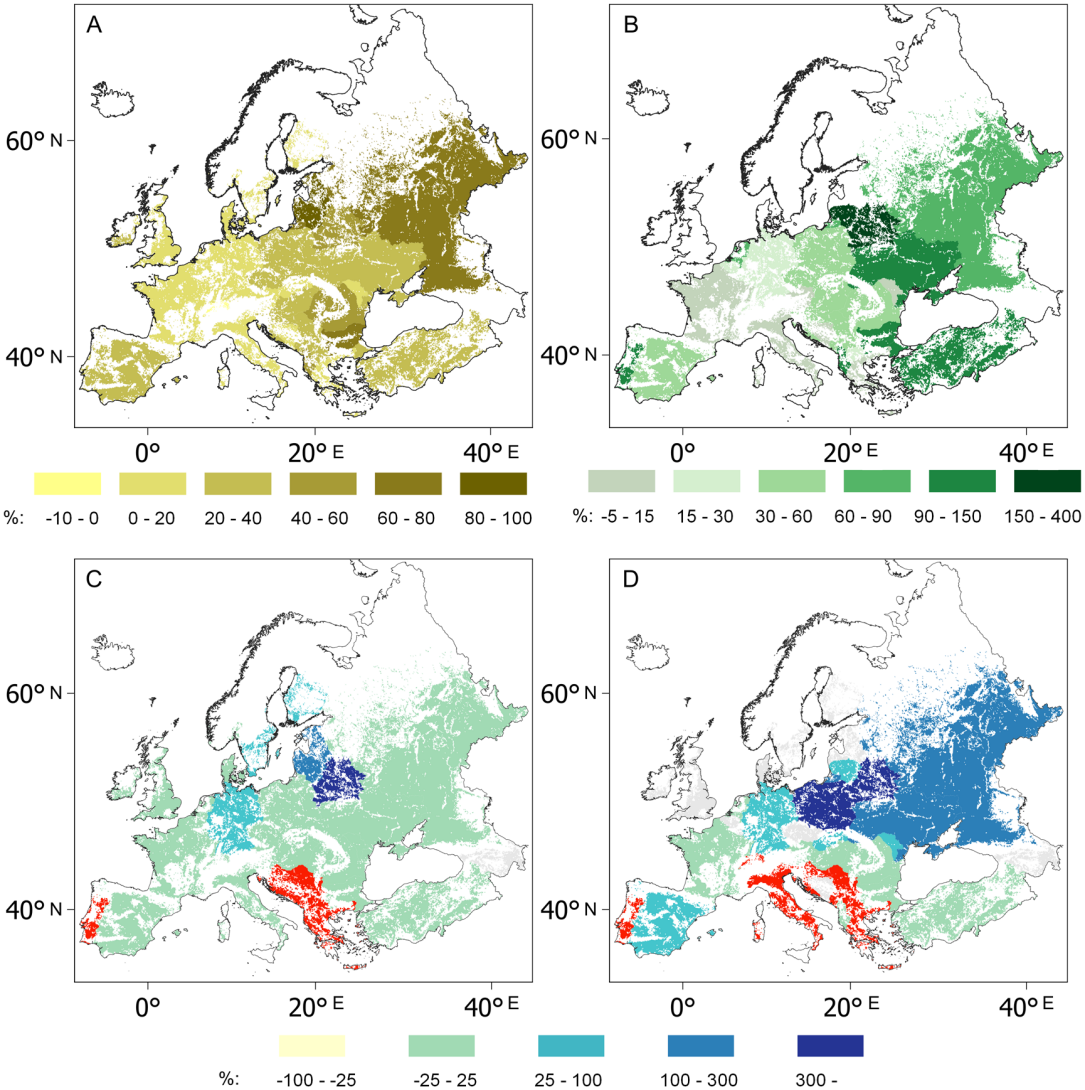
Country-by-country growth rates (%) in wheat (**A**) and maize yields (**B**), and wheat (**C**) and maize harvested areas (**D**) in Europe between 1993–1997 and 2013–2017 (1993–1997 = 100%). Data source: FAOSTAT^3^, blank map source: Eurostat GISCO^63^, software: QGIS 3.10^64^.

The European average of wheat and maize yields increased by an estimated 32%, and yields, with a few exceptions, increased significantly in the majority of European countries over the period of 1993–2017. In the case of wheat, however, this development was not continuous, since in accordance with global trends, the majority of the continent saw a yield stagnation or decline between the late 1980s and the mid-2000s (Fig. 2A, Supplementary Fig.S1). Although yield increase started in several countries and regions from the 2000s, wheat yield stagnation is observable over the entire period 1993–2017 in Austria, France, Moldova, the Netherlands, Norway, Portugal, Slovakia, Switzerland and the United Kingdom. Meanwhile, maize yield stagnated in Belgium, Bosnia–Herzegovina, France, Italy, Moldova and Slovakia between 1993 and 2017 (Fig. 3C, Supplementary Table S2, Fig.S2). Western Europe is one of the most efficient cereal producing regions on earth, where wheat and maize yields grew by 52% and 60%, respectively, comparing the periods of 1961–1991 and 1993–2017 (Table 1). No data could be found for cereal production of Eastern European countries (Belarus, Moldova, Russia and Ukraine) before 1992, making it difficult to track long-term development there. What is certain, however, is that a noticeable transformation took place from 1993–1997 to 2013–2017: wheat production leapt up, from 55 million tonnes per year (Mt y^−1^) to 95 Mt y^−1^, implying that what are now the four Eastern European countries (Table 1) are currently responsible for almost an eighth of global wheat production^3^. In this 72% increase one factor stands out, namely, yield improvement (Fig. 3A,C). Even more impressive indices may be found in the case of maize, since in Eastern Europe maize production increased sevenfold, from 6 to 42 Mt y^−1^ between 1993–1997 and 2013–2017 (Fig. 3C). As a result, two formerly negligible maize producers, Ukraine and Russia, have found a place among the ten world-leaders in maize production. The Ukrainian maize harvest (27 Mt y^−1^) was almost double that of France (14.7 Mt y^−1^), formerly the leading maize producer in Europe in 2013–2017, (Supplementary Table S6).

**Table 1:** Regional averages of the annual and May–August precipitation amounts, mean and maximum temperatures, and wheat and maize harvested areas, yields and total harvests in Europe, 1961–1990 and 1993–2017.

Period	Season	Europe	Western Europe^©^	Eastern Europe^‡^	Central Europe^§^	Southern Europe^◊^	Northern Europe^#^
**Precipitation, mm**							
1961–1990	Year	641	759	560	646	601	624
	May–Aug	226	259	247	278	132	248
1993–2017	Year	652	782	566	666	591	661*
	May–Aug	228	273	239	279	126	270*
**Mean temperature (°C)**							
1961–1990	Year	9.7	9.6	8.0	9.3	12.4	5.4
	May–Aug	17.6	15.7	18.0	17.5	19.6	14.4
1993–2017	Year	10.7*	10.6*	9.0*	10.2*	13.2*	6.5*
	May–Aug	18.7*	16.8*	19.2*	18.8*	20.8*	15.1*
**Maximum temperature (°C)**							
1961–1990	Year	14.4	13.8	12.3	12.7	18.6	9.6
	May–Aug	23.3	20.6	23.5	21.9	26.0	19.2
1993–2017	Year	15.3*	14.9*	13.3*	13.8*	19.6*	10.6*
	May–Aug	24.5*	21.9*	24.8*	23.3*	27.4*	20.1*
**Wheat**							
1961–1990	Harvested area, Mha		8.7		9.6	17.0	
	Yield, t ha^−1^		4.6		2.9	1.8	
	Total harvest, Mt		40.0		27.8	30.6	
1993–2017	Harvested area, Mha	66.0	10.8*	30.3	9.0*	13.8*	2.1
	Yield, t ha^−1^	3.4	7.2*	2.3	3.7*	2.6*	5.2
	Total harvest, Mt	226.7	77.8*	68.6	33.3*	35.9*	10.9
**Maize**							
1961–1990	Harvested area, Mha		1.8		7.6	2.6	
	Yield, t ha^−1^		5.2		3.4	4.0	
	Total harvest, Mt		9.4		25.8	10.3	
1993–2017	Harvested area, Mha	16.4	2.4*	4.0	7.7	2.3*	0.0
	Yield, t ha^−1^	5.2	8.9*	3.8	3.8*	8.5*	3.6
	Total harvest, Mt	85.4	21.4*	15.2	29.3*	19.5*	0.0

^©^Austria, Belgium, Denmark, France, Germany, Ireland, Netherlands, Switzerland and the United Kingdom; ^‡^Belarus, Moldova, Russia and Ukraine; ^§^Albania, Bosnia and Herzegovina, Bulgaria, Croatia, Czechia, Hungary, Montenegro, North Macedonia, Poland, Romania, Serbia, Slovakia and Slovenia (Yugoslavia 1961–1990) (Czechia and Slovakia fall under the term Czechoslovakia between 1961 and 1990); ^◊^ Greece, Italy, Portugal, Spain and Turkey; ^#^ Denmark, Estonia, Finland, Latvia, Lithuania, Norway, and Sweden (Lithuania alone represents Northern Europe in the case of maize between 1993 and 2017, since maize was cultivated only in this country during the entire period). Mha = million hectares, Mt = million tonnes. *Significant difference from 1961–1990 to 1993–2017 (*p* < .05) using Welsh t test. Data sources: CRU TS 4.04^62^, FAOSTAT^3^, Supplementary Tables S3, S4, S5 and S6.

Total wheat production almost doubled in Northern Europe, increased by about a third in Central Europe, by a fifth in Western and almost stagnated in Southern Europe between 1993–1997 and 2013–2017. Interestingly, maize appeared for the first time in the Danish national agro-statistics in 2010, and another Northern European country, Lithuania, showed the highest maize yield increase on the continent for the years 1993–2017 (Fig. 3C, Supplementary Fig. 2). The area under wheat and maize cultivation also grew by an estimated 1.3 and 1.1 million hectares in Western and Northern Europe, respectively, over this period. In contrast, the European part of the Mediterranean region, which covers the countries of Southern and South-Eastern Europe, lost an estimated 5.8 million hectares of wheat and maize fields between 1993 and 2017, and the scale of the loss between 1961 and 2017 may have exceeded 12 million hectares. According to FAO statistics, in these five decades, an estimated area of almost 19 million hectares of arable land was abandoned in the region^3^. This enormous degree of land abandonment was, however, accompanied by a modest yield increase generally, except in the Eastern Balkan countries, Bulgaria, Romania and Turkey.

### Climatic and economic factors

The annual and summer averages of maximum (Tmax) and mean (Tmean) air temperature increased significantly in every country and region of Europe but Ireland between the periods 1961–1990 and 1993–2017 (Table 1). Increase in annual Tmean and Tmax varied between 0.9 and 1.1 °C on the regional scale, and 0.5–1.3 °C (ΔTmean = 0.5–1.1 °C, ΔTmax = 0.6–1.3 °C) on the country scale comparing 1961–1990 to 1993–2017 (Table 1, Supplementary Table S3). A wider range (0.3 and 2.1 °C) was characteristic of the amount of seasonal change. The largest summer temperature increase occurred in Austria, Western Europe (ΔTmean_May-Aug_ = 2.1 °C and ΔTmax_July–August_ = 1.7 °C), while Fennoscandian winters warmed with the greatest intensity (ΔTmean_Jan–March_ = 1.3–1.9 °C) over the last 50 years (Supplementary Table S3). The warming trend was significant everywhere on the continent over the period of 1993–2017. By way of contrast, the amounts of annual and summer precipitation (Prec) have not changed significantly in any region of the continent over the last half century. At a higher spatial resolution, a trend-like increase of precipitation amounts can be discerned in most European countries, while they grew significantly in the countries of British Isles and Scandinavia. A non-significant decreasing trend appeared in the Southern European countries and Hungary, Central Europe (Supplementary Table S4). The highest annual precipitation sums were found in the Alps, and the West Balkan countries, as well as in Ireland and Norway, while the Eastern European countries and Finland got the lowest precipitation (Supplementary Table 1).

**Figure 4:**
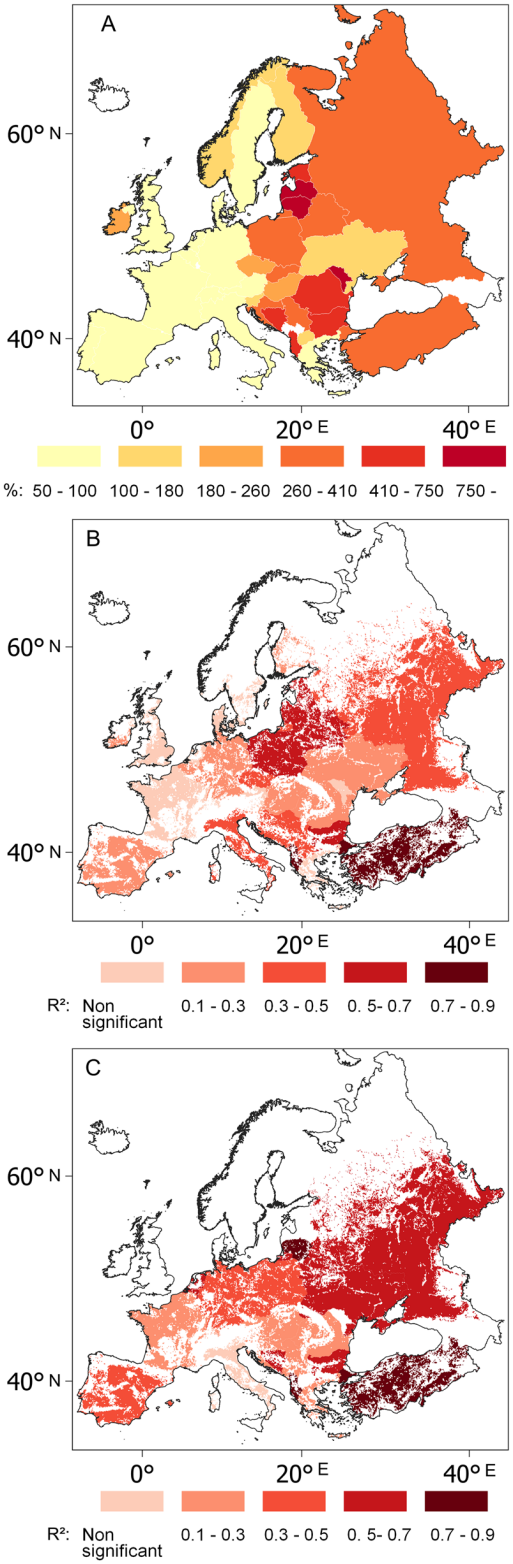
Country-by-country growth rates (%) of GDP per capita (**A**), with the coefficients of determination (R^2^) between national growth rates of GDP per capita and wheat (**B**) and maize yields (**C**) in the European countries in the period 1993–2017. Direction and significance of mean temperature trends in the European countries between 1993 and 2017. Calculations performed for 0.1° × 0.1° grid cells. N: negative direction; P: positive direction; S: significant; NS: non-significant. Data source: FAOSTAT^3^, World Bank^61^ and CRU TS 4.04^62^, blank map source: Eurostat GISCO^63^, software: QGIS 3.10^64^.

A growing GDP per capita characterised the entire continent comparing 1993–1997 to 2013–2017. While Southern and Northern Europe saw an estimated 73.7% and 104% per capita increase in GDP (taking the 1993–1997 mean as 100%), the Baltic and Balkan countries saw increases of 473% and a 309%, respectively (Fig. 4A). Similarly, a fourfold increase in GDP per capita was seen in the post-communist Eastern and Central European regions between 1993–1997 and 2013–2017. Meanwhile, in Western Europe, GDP per capita only doubled in the same period. A conspicuous East–West gradient may be observed in national growth rates of wheat and maize yields and GDP per capita (Figs. 3A,C and 4A).

### The associations between GDP per capita and cereal yields

The national growth rates of GDP per capita explained 69% of the variances in the yield growth rates of European wheat (*p* < 0.01; df = 31) and 26% in the case of maize (*p* = 0.01; df = 31) on a continental scale between 1993 and 2017, with the exception of two outliers (Lithuania and Moldova). On the country scale, significant associations between GDP per capita and cereal yields were found in the majority of countries for this period (Fig. 4B,C). The strongest GDP per capita-maize yield relationships occurred in the Baltic countries (R^2^ = 0.59–0.70; *p* < 0.01; *df* = 23 and 21), Albania (R^2^ = 0.86; *p* < 0.01; *df* = 23), Turkey (R^2^ = 0.74; *p* < 0.01; *df* = 23) and Belarus (R^2^ = 0.66; *p* < 0.01; *df* = 23) and strong GDP-yield associations appeared in the big Eastern European producers, too (R^2^_Ukraine_ = 0.60; R^2^_Russia_ = 0.56; *p* < 0.01, *df* = 23 ) . As for the biggest cereal producer, GDP increase explained 37% of the variances of wheat yield change in Russia during 1993–2017.

### Temperature, precipitation and cereal yield associations

**Figure 5:**
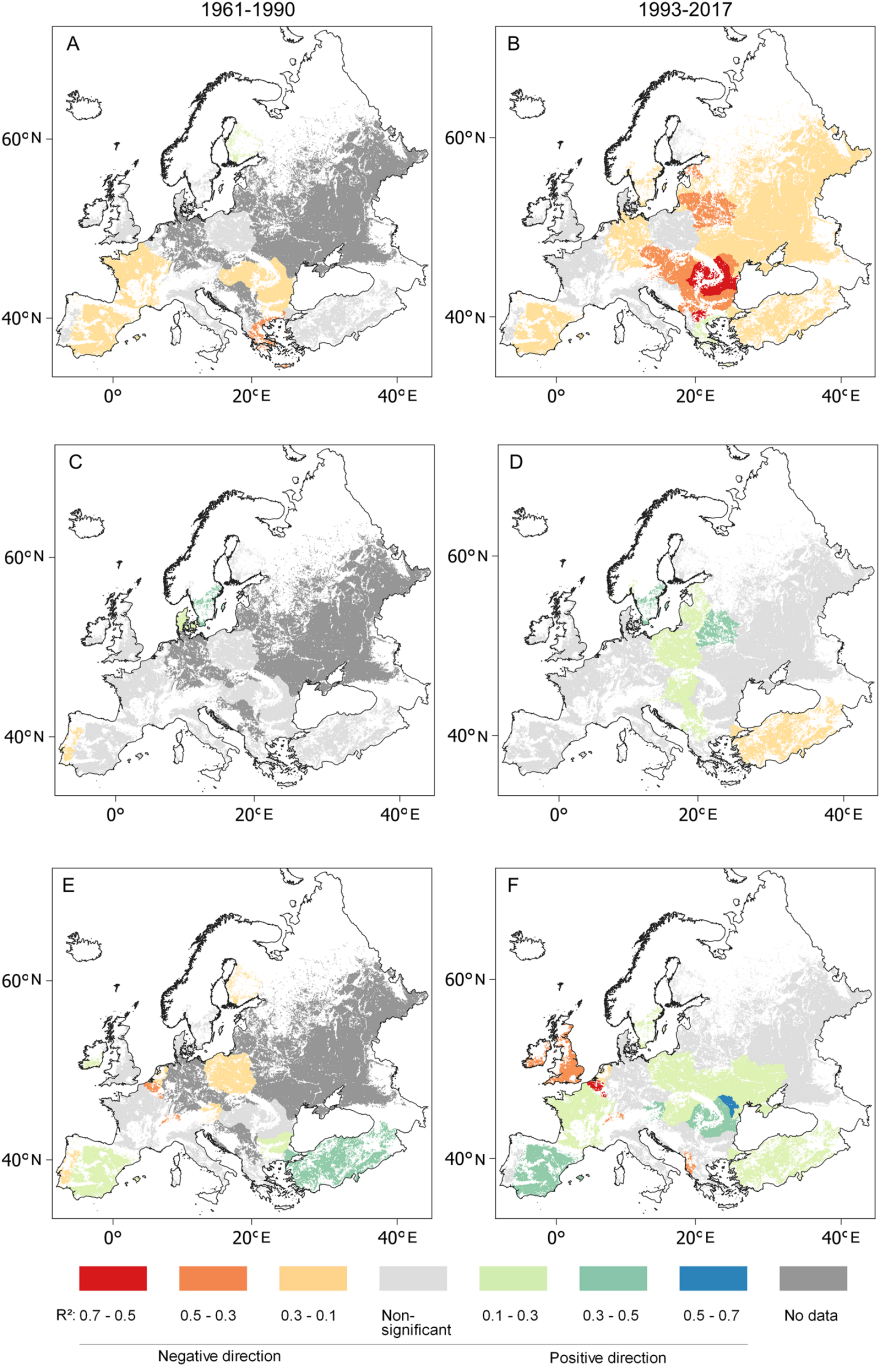
Coefficients of determination (R^2^) between the first-differences of May–July mean temperature and wheat yields for (**A**) 1961–1990 and (**B**) 1993–2017; between the first-differences of January–March mean temperature and wheat yields for (**C**) 1961–1990 and (**D**) 1993–2017; between the first-differences of September–July precipitation sums and wheat yields for (**E**) 1961–1990 and (**F**) 1993–2017 in European croplands. Data source: FAOSTAT3 and CRU TS 4.04^62^, blank map source: Eurostat GISCO^63^, software: QGIS 3.10^64^.

**Figure 6:**
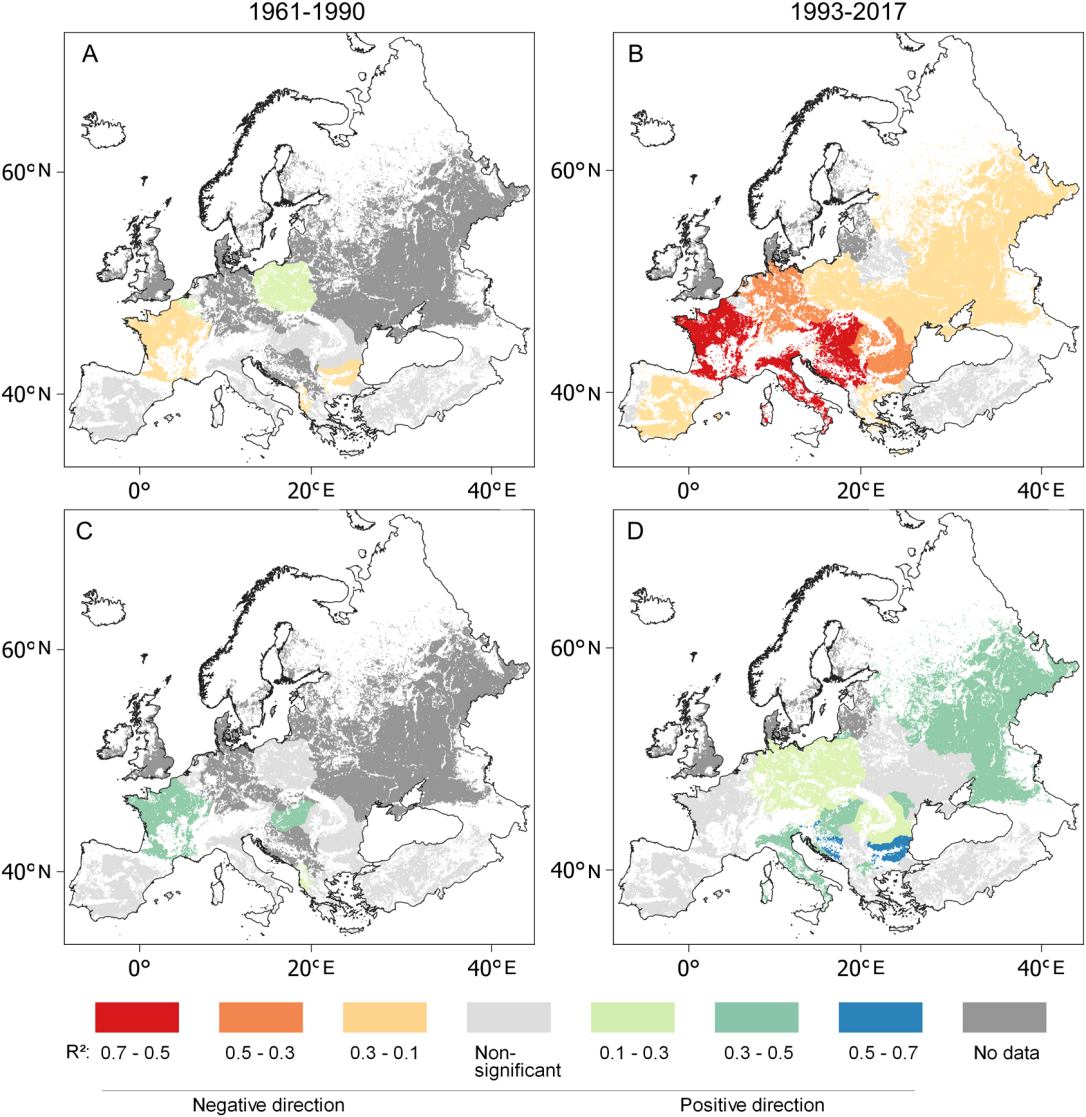
Coefficients of determination (R^2^) between the first-differences of July–August max temperature and maize yields for (**A**) 1961–1990 and (**B**) 1993–2017, as well as between May–August precipitation sums and maize yields for (**C**) 1961–1990 and (**D**) 1993–2017 in European croplands. Data source: FAOSTAT3 and CRU TS 4.04^62^, blank map source: Eurostat GISCO^63^, software: QGIS 3.10^64^.

**Figure 7:**
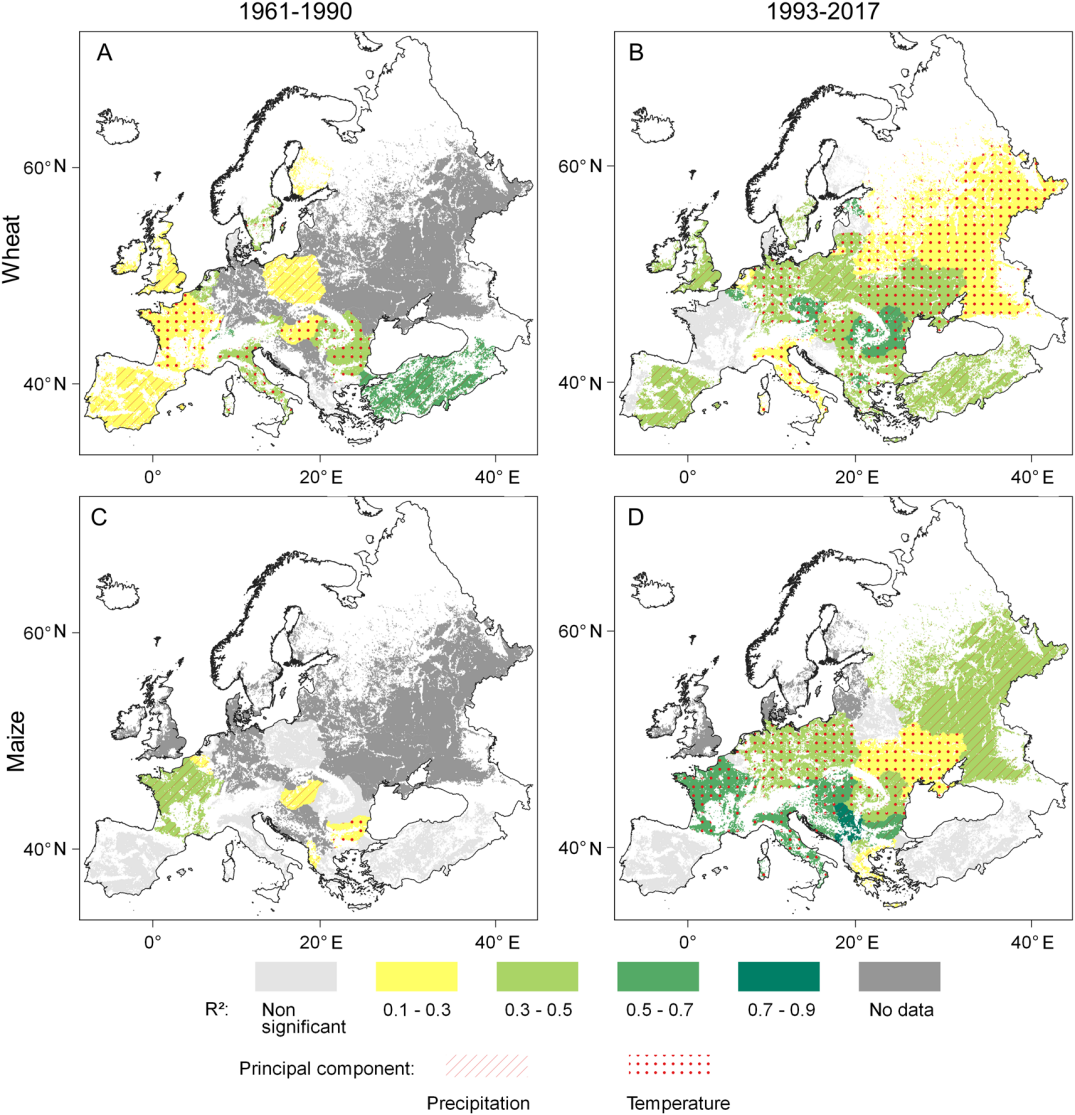
Coefficients of determination (R^2^) between the first-differences of the combined explanatory factors of the averages of May–July mean temperature and September–July precipitation sum and wheat yields for (**A**) 1961–1990 and (**B**) 1993–2017, as well as between the combined explanatory factor of the averages of July–August max temperature and May–August precipitation sums and maize yields for (**C**) 1961–1990 and (**D**) 1993–2017 in European croplands. Data source: FAOSTAT^3^ and CRU TS 4.04^62^, blank map source: Eurostat GISCO^63^, software: QGIS 3.10^64^.

The period of 1961–2017 brought a spectacular intensification in linear associations between climatic and cereal yield variances in Europe (Figs. 5, 6, 7). May–July mean temperature showed a significant relationship to wheat yields, and this was in a negative direction in the case of 49% of European wheat fields between 1961 and 1990. Then, in the period 1993–2017, however, the area in which this significant negative relation grew to 78% of the area under wheat cultivation (Fig. 5A,B).

The greatest degree of sensitivity of wheat yields to increasing summer temperature (Table 1) was found in Romania (R^2^ = 0.63; *p* < 0.01; df = 22), and also in the neighbouring countries, indicating the presence of a sensitive zone of wheat cultivation in Central and Eastern Europe in the period of 1993–2017 (Fig. 5B). Interestingly, the significant negative relationship between May–July temperature and wheat yield observed for the period of 1961–1990 in France and Greece had disappeared by 1993–2017. May–July mean temperature accounted for an estimated 22% of wheat yield variances over the period of 1993–2017 (R^2^_weighted by harvested area_ = 0.25; R^2^_weighted by total production_ = 0.22; R^2^_range_ = 0.14–0.63) in ten big wheat producers on the continent (Russia, Germany, Ukraine, Turkey, Romania, Italy, Spain, Bulgaria, Hungary, Czechia and Serbia), where almost two-thirds of European and a quarter of global wheat was produced between 2013 and 2017. An increasing positive impact of January–March Tmean on wheat yields was observed in 9.5% of European areas under wheat cultivation in Northern and Central Europe comparing 1961–1990 to 1993–2017 (Fig. 5). A significant relationship between January–March Tmean and wheat yield appeared in Denmark in 1961–1990 (R^2^ = 0.22; *p* = 0.01; df = 22), though this vanished over the period of 1993–2017. Meanwhile, the negative direction observed in the association between September–July precipitation and wheat yield switched to a positive one almost everywhere between 1961–1990 and 1993–2017, except in countries in the wettest regions (the British Isles, Low Countries, Switzerland and Albania) (Fig. 5e,f).

Similarly, an increase in the positive impact of May–August precipitation on maize yields was observed in many countries of the continent (Fig. 6C, D). The countries where a significant positive relationship was found between 1961 and 1990 covered an estimated 30.5% of European areas of maize cultivation, but this positive precipitation yield relationship was found in countries covering 63.7% of European maize fields between 1993 and 2017 (Fig. 6C,D). The temperature maize yield association was characterised by a more dynamic transformation. The positive relationships obtaining between July–August Tmax and maize yield in the 1961–1990 period had disappeared everywhere by 1993–2017. In the meanwhile, the areal extent of the significant negative impact of July–August Tmax on maize yields found in 23% of European maize cultivation came to extend across almost the entire (94%) continent (Figs. 6A,B). The tendency observed is alarming, since the average of temperature variables increased significantly between the periods 1961–1990 and 1993–2017 (Table 1). July–August maximum temperature accounted for an estimated 40% of the variance in annual maize yield over the period 1993–2017 (R^2^_weighted by harvested area_ = 0.43; R^2^_weighted by total production_ = 0.40; R^2^_range_ = 0.00–0.67) in the ten biggest maize producers on the continent (Ukraine, France, Russia, Romania, Hungary, Italy, Serbia, Turkey, Spain, Germany and Poland), where almost 80% of European and 10% of global maize production took place.

Both the strength and the spatial validity of linear associations have increased between the combined temperature/precipitation climatic factor and wheat and maize yields in Europe over the period 1961–1990. As a result of this process, Central European wheat and maize yields was found to have the highest degree of climate sensitivity on the continent (Fig. 7). Overall, the combined temperature-precipitation predictor has proven capable of explaining between 12 and 67% of year-to-year changes in wheat yields, and this association was found to be significant for 90% of European areas under wheat cultivation during the period of 1993–2017. The combined climatic factor accounted for between 24 and 81% of maize yields in 92% of the European areas under maize cultivation. Within the combined (temperature-precipitation) climatic variable, temperature and precipitation were identified as the principal factor in the case of 65% and 25% of the wheat growing areas, respectively, over the period of 1993–2017 (Fig. 7B). Almost the same spatial ratio was arrived at for temperature and precipitation as the primary explanatory factor in the case of maize yield regressions, too. This temperature—precipitation spatial ratio ranged from 43 to 53% in the case of wheat and from 6 to 31% in the case of maize during the 1961–1990 period (Fig. 7A,C).

## Discussion

The dynamic growth in the global average of grain production (annual 1.4% per capita) brought about by the Green Revolution of the 1950s and 1960s slowed down between the 1980s and the 2000s (annual 0.6% per capita). Now, however, the current trend towards increasing production (an annual 1.6% per capita) harks back to the glory days of the Green Revolution, seemingly refuting the earlier gloomy predictions^63^ that the forecast 3 billion tonnes annual grain demand for 2050 neither could nor would be satisfied. The spectacular development of global cereal production was mainly explained by dynamic efficiency gains accompanied by a relatively slow increase of harvested areas (Fig. 1). This positive development in global food security strongly challenges the validity of former bleak prospect that was raised at the end of an almost two-decade stagnation of yield and production trends in key areas of cereal production^8,21,28^. The geographically varying drivers behind the transformations underline the notion that global analyses would benefit from and be complemented by in-depth analyses of regional patterns.

A profound realignment of the regional arrangement has been observed in European wheat and maize production over the period of 1993–2017. Two spatial processes have largely determined the direction of the transformation: (1) while the agro-technological advancement of Eastern Europe has pulled the focus of European cereal production from West to East, (2) a warming climate has placed pressure on growing areas. The resultant vector of these two factors seems to indicate the north-eastern sector of the European grain chessboard, the Baltic states as the main beneficiaries of recent transformations (Figs. 2, 3 and 4). Indeed, the highest growth rates in wheat yield were observed in Latvia (92%), followed by Estonia (87%) and Lithuania (86%) from 1993–1997 to 2013–2017 (Fig. 2A, Supplementary Fig. S1). The highest increases in maize yield were observed in Lithuania (385%) and Belarus (213%) (Supplementary Fig. S2). In this region, the greatest increase in yield on the continent was accompanied by the greatest increases in GDP, a factor which explains an estimated 61 to 88% of the increases in wheat and maize yields between 1993 and 2017 (Fig. 4). The shift in habitat range for many plant species is a corollary of climate change^64,65^. Due to the warming climate, maize has appeared in the agricultural statistics of northern countries where it had not been previously cultivated^3^. Countries which had traditionally been maize importers, like Poland and Russia, have become major exporters.

Although the “invasion of Eastern European grain” may appear to be a new phenomenon, this is only true in the context of modern times; what is, in fact, happening is that a 3000-year-old grain production-consumption system is being restored before our eyes. The two main elements of this system are a food surplus in the plains surrounding the Black Sea Basin and food demand in the Mediterranean Region. This structure was discovered and developed by the ancient Greeks and it lasted, with some interruptions, until the end of the empire of the Russian Tsars^13,66,67^. With the collapse of Romanov Russia, food exports from Eastern Europe disappeared from the market, and the Soviet regime was not able to achieve a stable and lasting self-sufficiency in food. The almost complete isolation of the Soviet bloc countries from international technological development and markets, the distorted terms of trade between communist countries, the low level of capitalization of the entire region, the collectivisation of lands and capital goods that acted as a demotivating factor on labour increased the long-term East–West divide of Europe. The degree of technological backwardness became extreme in certain cases before the collapse of communist regimes. From the Eastern European regime changes of 1989 and after, an almost century-long interruption ended. A rapid technological transfer lurched from West to East and the “natural” flow of food from the Black Sea Basin to the Mediterranean is being re-established. As a result, post-communist Eastern and Central European countries have come to occupy high positions in rankings of global exporters. The outlined food production-consumption system has emerged as a part of long-term historical structures and worked under peace times over the last 3000 years when the life took place in “normal operation”. Wars, like the Ottoman-Russian wars in the 18th century, Crimean War in the 1850s, the Russian invasion in Ukraine today, or other stochastic phenomena such as the irrational communist regime in the Sovietunion can block the operation of the production-consumption system.

The loser in the big regional transformation is the European part of the Mediterranean region, including Southern Europe and the Balkan Peninsula. There, the area of wheat and maize cultivation declined by an estimated 12 million ha between 1961 and 2017, and the rate of shrinkage has accelerated in the first decades of the twenty-first century. The abandonment of cereal fields was a part of a wider landscape transformation in the European part of the Mediterranean, since almost 19 million ha of arable land was abandoned here over the period 1961–2017^3^. The extent of the deserted arable lands in this region was equivalent to an estimated two-thirds of the arable lands in Australia (31 million ha) in 2017^3^. For instance, the cultivated area of the two grains disappeared almost completely in Portugal. The harvested area of wheat was reduced to half of its former extent in Italy and Spain and decreased by a third in Greece from 1961 to 2017. The harvested area of maize also shrank to half in Italy, to two-thirds in Greece and three-quarters in Spain in this period. The rather low wheat yields did, however, display a modest development curve in the region. Conversely, maize yields in Southern Europe reached almost 10 t ha^−1^, one of the highest figures for global maize production. Turkey also belongs to this group in which maize yields have skyrocketed (Figs. 2B and 3B). The reason for this difference in yield trends in the region is that maize producing areas have been irrigated in a major part of these countries, too, but the importance of wheat, with its lower yield potential and water use efficiency, has diminished in Mediterranean irrigation programs^68–70^. This adaptation strategy illustrates that the challenges of a warming climate and shrinking freshwater resources have narrowed the opportunities for cropland farming in drylands, including the Mediterranean climate zone, where farming depends more and more on irrigation. The accelerating dynamics of environmental crisis discovered here^37^ as well as in model-based predictions^19,21^ suggest that the significant shrinkage in the area under cereal cultivation will continue in this region. Such a rate of cropland loss as this, in turn, highlights the European part of the Mediterranean Region as a hotspot in terms of global food security issues.

Reviewing the climate impacts on wheat and maize yields set forth here, the positive Tmean and Tmax—yield relationships observable in the period 1961–1990 had disappeared everywhere by 1993–2017, while on the entire continent only negative temperature—yield relationships could be found for 1993–2017. The highest degree of sensitivity of wheat and maize yields to increasing summer temperature was found in Eastern and Central Europe. These regions, however, are the centres of the development of crop production in Europe (Table 1). The major part of the European yield gap, i.e. growing potential, was also identified as being located in these regions^7,44^. In line with previous research^71,72^, the results presented here also constitute a warning in relation to this option. The main conflict zone of the economic and climatic lines of force lay in the southern region of the continental zone, including Moldova. Here, a remarkably large increase in GDP (354%) was observed between 1993–1997 and 2013–2017 (Fig. 4A), while at the same time, the vulnerability of wheat and maize yields to climatic factors was estimated to be the highest on the continent (Figs. 5, 6 and 7), and the already low maize yields decreased further over that time (Fig. 3B, Supplementary Fig. S2). An examination of the outlier records does, however, serve as a warning to avoid any monocausal or oversimplified explanation, indicating the requirement for a more complex examination of the interconnected environmental and social factors behind these rapid and profound regional transformations.

Besides negative effects, a positive impact of climate change must also be mentioned, since increasing January–March mean temperatures have had a significantly positive impact on wheat yields in Central and Northern Europe. This development is also in line with the results of recent research^35^ indicating that high latitude frost-prone agricultural zones will benefit from a warming climate. The novelty of this analysis, however, is that it could be demonstrated that this positive impact of climate change has already begun and has indeed been observable in extensive regional patterns over recent decades. Another fresh result is that this positive impact can be identified across a wide zone from Northern Europe to the Northern Balkan Peninsula, overlapping with the Central and Eastern European regions, where the greatest vulnerability of wheat yields to increasing May–July temperature was also identified (Figs. 5A,B, 7A,B). The results presented here suggest that the positive impact of warming January–March temperatures might have compensated for a certain part of the negative impact of increasing May–July mean temperature on wheat yields in this area (Fig. 5A–D).

## Method

Putting the research into a broader geographical context, the long-term trends in global cereal production have been presented. For this, World Bank annual cereal production and the harvested areas of cereals and population data^61^ for the period of 1961–2017 were used. World Bank data for cereal production was based on FAO datasets^30^, in which crops harvested for dry grain are classified as cereals, and fodder as well as industrial crops are excluded^73^. Then, monthly meteorological time series for the 1961–1990 (30-year) and 1993–2017 (25-year) periods were extracted from the CRU TS 4.04 (land) Tmean, Tmax, Prec, minimum temperature (Tmin), the Palmer Drought Severity Index (PDSI)^74^ and the Standardised Precipitation-Evapotranspiration Index (SPEI1 and SPEI3)^75^ climatic variables using the Climate Explorer managed by the Royal Netherlands Meteorological Institute (KNMI)^62,76^. National yield averages and harvested areas were obtained from the datasets of the FAO^3^ for the same time periods, while the World Bank’s estimates of gross domestic product (GDP) per capita of European countries were used for the period between 1993 and 2017. The 1993–2017 timeframe was selected since a great number of currently important grain producing European countries attained their status as independent nations in the early 1990s. To mitigate the power of outliers of certain years 5-year averages (1993–1997, 2003–2007 and 2013–2017) were used in the analysis and visualisation of growth rates (%) and the changes on yield rank tables for the period 1993–2017 (Fig. 2). While Turkey and Russia were included in the analysis, the smallest countries, for which data were not available in the FAO datasets, were excluded. The regional classification of the countries followed the FAO and USDA protocol^77^.

For an explicit spatial analysis, croplands were identified and delineated as 0.5° × 0.5° grid cells in which at least 20% of the cell area is covered by croplands in the Earthstat cropland dataset (at a resolution of 5 arc min)^78^. Using the selected grid cells of the climatic variables that overlap with croplands, spatial averages of monthly Tmean, Tmax and Prec were calculated for each country and region for the two periods. Using the first-differences method, the variables under examination were detrended. The detrended spatially explicit averages of climatic variables were then correlated with national averages of wheat and maize yields using ordinary and multiple linear regression and nonparametric bootstrap resampling methods^79,80^ for different vegetation periods. The visualisation of yield trends was carried out using slope graphs and locally weighted regression generated by the CGPfunctions^81^ and devtools packages in an R environment^82^. Trends were evaluated via Mann- Kendall tests using the kendall package^83^.

Climatic parameters affect plant and seed development in different ways in each phenological growth stage of cultivated wheat species^84^. But the fact that there are no separate long-term data on yields of winter and spring wheat (*Triticum aestivum*), durum wheat (*T. durum*) or spelt (*T. spelta*) does complicate the precise definition of vegetation periods. The estimated amount of winter wheat as a percentage of all wheat sown in Europe is above 95% for the 2002–2017 period^60^. In Czechia, Ireland, Latvia, Lithuania, the Netherlands, Poland and Sweden the share of winter wheat ranged between 75 and 93%, but the share of spring wheat is high in Finland (87%) and Norway (40%)^60^. The life-cycle of winter wheat spans the period September/October–June/July, while spring wheat spans the months between March/April and September/October. The share of durum and spelt in the total wheat harvest is minor in every European country. September/October–March/April is the period of soil moisture recharge, characterised by a mostly positive soil water balance (precipitation > evapotranspiration), and marks the period when wheat is vulnerable to soil saturation (i.e. stress caused by poor soil aeration). Later, between April and July, however, depending on the soil water balance, the direction of the relationship between Prec and yield variances is likely to be positive^27^. This research therefore focuses on the statistical associations between the September–July precipitation sums, with the May–July mean temperature, PDSI, SPEI 1 and SPEI3 as independent variables, and annual wheat yields as dependent variables. It was also reasonable to suppose that any winter and early spring temperature increase would have a positive effect on yields of winter wheat in northern part of the European cropland areas^26^ comparing 1961–1990 to 1993–2017, and it was for this reason the linear relationship between the variables of Jan–March Tmean, Tmin and annual wheat yields was also examined. In the case of maize, the response of yield to the Tmean and Prec variables was examined for the entire period of active root water uptake (May–August), but Tmax, PDSI, SPEI 1 and SPEI3 were analysed for the high summer months (July and August). Of the climatic factors examined, those which explained the highest rate of yield variances Tmean, Tmax and Prec were selected for the detailed analysis and interpretation in the main text. The deterministic coefficients of the associations of Tmin-wheat yield, PDSI-wheat and maize yield and the SPEI1-wheat and maize yield and SPEI3-wheat and maize yield may be found in the Supplementary Table S9–S15. To detect the impact of technological development between 1993 and 2017, the association between the ratio of changing GDP per capita as an explanatory variable and the ratio of changing wheat and maize yields as response variables was determined with the use of linear regression tests. Here, it should be mentioned that the year-to-year complex index of Total Factor Productivity (TFP) in agriculture^49^ and fertiliser use in the European countries was intended for use in the analysis of the impact of technological development on yield changes. The available USDA TFP and FAO fertiliser data, however, was found to contain such serious inaccuracies and internal inconsistencies (Supplementary Table S8) that these indices proved unsuitable for the intended scientific analyses.

## Supplementary Information

Supplementary Information.

## Data Availability

All country scale year-to-year data and R codes are available on the Open Science Framework (OSF; https://doi.org/10.17605/OSF.IO/W6TZN). All country scale averages and test results are available in the Supplementary information of the online version of this article. These data can be used to reproduce all analyses. All other relevant data are available from the corresponding author on request.
