# Supplementary material for: Climate change and modernization drive structural realignments in European grain production
Source: Sci Rep. 2022 May 5;12:7374. doi: 10.1038/s41598-022-10670-6 (PMC9072400; doi:10.1038/s41598-022-10670-6)
Supplement: Supplementary file 1 — Supplementary Information. [file 41598_2022_10670_MOESM1_ESM.pdf]

Supplementary information

Tectonic shift on the European grain chessboard

Table S1 Shapiro-Wilk’s test results for the normal distribution of the analysed variables in the European countries for 1993–2017 and 1961–1990

Country	1993–2017						1961–1990				
	Wheat	Maize	GDP per capita	Tmean	Tmax	Prec	Wheat	Maize	Tmean	Tmax	Prec
						p					
Albania	0.01	0.06	<0.01	0.76	0.94	0.74	0.05	0.09	0.98	0.52	0. 37
Austria	0.71	0.41	<0.01	0.64	0.71	0.54	0.30	0.14	0.97	0.66	0.01
Belarus	0.37	0.69	<0.01	0.79	0.20	0.64					
Belgium	0.73	0.14	<0.01	0.77	0.52	0.48	0.14	0.06	0.13	0.05	0.74
Bosnia	0.99	0.47	<0.01	0.40	0.59	0.47					
Bulgaria	0.41	0.71	<0.01	0.70	0.45	0.75	0.23	0.52	0.67	0.57	0.18
Croatia	0.70	0.49	<0.01	0.29	0.37	0.89	0.02				
Czechia	0.01	0.90	<0.01	0.94	0.98	0.53					
Denmark	0.76		<0.01	0.92	0,26	0.25	0.02		0.02	0.47	0.45
Estonia	0.16		<0.01	0.11	0.82	0.15					
Finland	<0.01		0.02	0.31	0.26	0.87	0.49		0.78	0.99	0.28
France	0.20	0.78	0.01	0.93	0.72	0.33	0.35	0.26	0.05	0.33	0.89
Germany	0.99	0.52	0.02	0.49	0.52	0.28					
Greece	0.90	0.44	0.10	0.37	0.55	0.52	0.17	<0.01	0.99	0.53	0.56
Hungary	0.80	0.25	<0.01	0.33	0.61	0.83	0.16	0.15	0.62	0.65	0.25
Ireland	0.99		0.04	0.12	0.14	0.05	0.01		0.17	0.11	0.52
Italy	0.37	0.26	0.02	0.21	0.73	0.28	0.71	0.02	0.30	0.48	0.37
Latvia	0.21		<0.01	0.73	0.64	0.28					
Lithuania	0.71	0.01	0.01	0.95	0.45	0.87					
Moldova	0.97	0.67	<0.01	0.83	0.63	0.63					
Netherlands	0.31	0.68	0.01	0.70	0.72	0.98	0.02	<0.01	0.69	0.09	0.43
North Macedonia	0.62	0.01	<0.01	0.38	0.52	0.63					
Norway	0.68		0.01	0.86	0.68	0.97	0.95		0.23	0.26	0.98
Poland	0.21	0.63	<0.01	0.86	0.33	0.28	0.30	0.02	0.08	0.32	0.28
Portugal	0.74	0.04	0.01	0.40	0.65	0.22	0.53	<0.01	0.93	0.14	0.01
Romania	0.70	0.69	<0.01	0.55	0.69	0.93	0.40	0.43	0.17	0.75	0.96
Russia	0.49	0.51	0.01	0.79	0.83	0.37					
Serbia	0.87	0.64	<0.01	0.40	0.84	0.92					
Slovakia	0.74	0.22	<0.01	0.22	0.86	0.05					
Slovenia	0.74	0.91	0.01	0.42	0.63	0.32					
Spain	0.56	0.02	0.01	0.30	0.34	0.14	0.01	0.02	0.69	0.01	0.39
Sweden	0.06		0.01	0.95	0.24	0.44	0.67		0.03	0.35	0.73
Switzerland	0.05	<0.01	<0.01	0.03	0.45	0.13	0.49	0.96	0.05	0.66	0.76
Turkey	0.25	0.01	<0.01	0.69	0.18	0.33	0.02	<0.01	0.36	0.83	0.74
Ukraine	0.51	0.05	0.01	0.71	0.40	0.39					
United Kingdom	0.93		0.09	0.84	0.15	0.88	0.02		0.14	<0.01	0.17

p-value > 0.05 indicating that the distribution of the records not significantly differs from normal distribution. Tmean = May–August temperature mean, Tmax = July–August maximum temperature, Prec = May–August precipitation sum.

Table S2 Mann-Kendall test results of wheat and maize yield trends in the European countries for 1993–2017

Country	Wheat		Maize	
	tau	*p*	tau	*p*
Albania	0.66	< 0.01	0.89	< 0.01
Austria	0.11	0.43	0.41	< 0.01
Belarus	0.53	< 0.01	0.77	< 0.01
Belgium	0.47	< 0.01	0.27	0.06
Bosnia	0.29	0.04	0.11	0.44
Bulgaria	0.62	< 0.01	0.61	< 0.01
Croatia	0.55	< 0.01	0.47	< 0.01
Czechia	0.47	< 0.01	0.50	< 0.01
Denmark	0.33	0.02		
Estonia	0.70	< 0.01		
Finland	0.40	< 0.01		
France	0.02	0.19	0.21	0.15
Germany	0.44	< 0.01	0.57	< 0.01
Greece	0.32	0.02	0.59	< 0.01
Hungary	0.36	0.01	0.36	0.01
Ireland	0.47	< 0.01		
Italy	0.53	< 0.01	0.15	0.29
Latvia	0.78	< 0.01		
Lithuania	0.69	< 0.01	0.76	< 0.01
Moldova	0.03	0.86	0.17	0.25
Netherlands	0.23	0.10	0.55	< 0.01
North Macedonia	0.36	0.01	0.41	< 0.01
Norway	0.15	0.29		
Poland	0.73	< 0.01	0.52	< 0.01
Portugal	0.23	0.10	0.81	< 0.01
Romania	0.43	< 0.01	0.37	0.01
Russia	0.62	< 0.01	0.63	< 0.01
Serbia	0.42	< 0.01	0.44	< 0.01
Slovakia	0.13	0.35	0.28	0.05
Slovenia	0.43	< 0.01	0.43	< 0.01
Spain	0.36	0.01	0.84	< 0.01
Sweden	0.38	0.01		
Switzerland	0.11	0.43	0.47	< 0.01
Turkey	0.75	< 0.01	0.80	< 0.01
Ukraine	0.31	0.03	0.78	< 0.01
United Kingdom	0.25	0.07		

p-value < 0.05 indicating that the trend of changes was significantly consistent. The sign of tau-value indicates the direction of the change, while the measure of tau-value the gradient of the trend line.

Table S3 Annual and May–August temperature means in the croplands of the European countries in 1993–2017 and 1961–1990. Data are means ± SD.

Country	Mean temperature, °C					
	1993–2017			1961–1990		
	Year	May-Aug	Jan–March	Year	May-Aug	Jan–March
Albania	12.4*±0.2	20.0*±0.3	4.7±0.6	11.6±0.1	18.6±0.3	4.2±0.9
Austria	9.7*±0.3	18.0*±0.3	1.6*±0.8	9.2±0.3	15.9±0.7	0.5±0.9
Belarus	7.4±0.35	17.2±0.4	-2.5±1.0			
Belgium	10.8*±0.3	16.9*±0.2	4.7*±0.7	9.6±0.3	15.7±0.4	3.4±0.9
Bosnia	10.5±0.3	18.7±0.4	2.3±0.8			
Bulgaria	12.0*±0.3	21.0*±0.3	2.9*±0.8	11.1±0.2	19.6±0.25	2.0±0.7
Croatia	12.0±0.3	20.3±0.4	3.9±0.8			
Czechia	8.9±0.3	17.0±0.3	1.0±0.8			
Denmark	8.8*±0.3	15.0*±0.3	2.6*±0.8	7.8±0.3	14.2±0.3	0.7±1.1
Estonia	6.0±0.3	15.1±0.4	-3.6±1.0			
Finland	4.4*±0.4	13.9*±0.4	-5.6*±1.0	3.3±0.5	13.3±0.4	-7.2±1.5
France	12.1*±0.2	18±0.25	6.2*±0.5	11±0.3	16.8±0.4	5.2±0.7
Germany	9.6±0.3	16.7±0.3	2.5±0.8			
Greece	14.3*±0.2	22.0*±0.3	6.5±0.5	13.7±0.2	20.7±0.2	6.2±0.4
Hungary	11.2*±0.3	20.0*±0.4	2.5*±0.8	10.1±0.3	18.6±0.3	1.4±1.0
Ireland	10.0*±0.2	13.7*±0.2	6.2*±0.4	9.4±0.2	13.2±0.3	5.5±0.5
Italy	13.3*±0.2	20.5*±0.3	6.1±0.5	12.3±0.1	19±0.3	5.4±0.5
Latvia	6.7±0.3	15.6±0.4	-2.5±1.0			
Lithuania	7.3±0.3	16.2±0.4	-1.8±1.0			
Moldova	10.6±0.3	20.6±0.4	0.6±0.9			
Netherlands	10.2*±0.3	16.0*±0.3	4.3*±0.7	9.1±0.3	14.9±0.4	3.0±1.0
North Macedonia	10.8±0.2	19.4±0.3	2.2±0.6			
Norway	6.7*±0.4	14.1*±0.4	-0.9*±0.8	5.8±0.3	13.5±0.3	-2.2±1.3
Poland	8.8*±0.3	17.0*±0.3	0.6*±0.9	7.9±0.4	16.0±0.4	-0.5±1.2
Portugal	15.7*±0.2	21.1*±0.3	10.2*±0.8	14.9±0.2	20.0±0.4	9.6±0.3
Romania	10.7*±0.3	20.0*±0.3	1.4*±0.8	9.8±0.5	18.6±0.3	0.3±1.0
Russia	6.6±0.3	18.8±0.5	-5.9±1.0			
Serbia	11.2±0.3	19.9±0.4	2.4±0.8			
Slovakia	9.6±0.3	18.2±0.3	1.1±0.8			
Slovenia	7.7±0.3	15.3±0.4	0.1±0.7			
Spain	14.4*±0.2	21.2±0.3	8.0*±0.4	13.6±0.2	20.0±0.4	7.3±0.3
Sweden	7.1*±0.3	14.1*±0.4	-0.6*±0.8	6.2±0.4	13.5±0.3	-1.9±2.2
Switzerland	9.2*±0.3	16.2*±0.3	2.3*±0.6	8.1±0.2	14.9±0.3	1.3±0.7
Turkey	12.0*±0.3	20.4*±0.2	2.9±0.6	11.3±0.2	19.4±0.2	2.3±0.7
Ukraine	9.3±0.3	19.6±0.4	-1.1±0.9			
United Kingdom	9.7*±0.2	14.4*±0.2	5.0*±0.5	8.9±0.2	13.6±0.3	4.0±0.6

*Significant difference from 1961–1990 to 1993–2017 ($p < .05$) using Welch t-test. SD = standard deviation.

Table S4 Annual and May–August means of maximum temperature and precipitation sums in the croplands of the European countries in 1993–2017 and 1961–1990. Data are means ± SD.

Country	1993–2017				1961–1990			
	Maximum temperature, °C		Precipitation, mm		Maximum temperature, °C		Precipitation, mm	
	Year	Jul-Aug	Year	May-Aug	Year	Jul-Aug	Year	May-Aug
Albania	18.4*±0.3	28.9*±0.5	1064.4±75.8	193.9±22.6	17.7±0.2	27.4±0.5	1055.6±79.3	201.5±30.6
Austria	15.2*±0.4	25.1*±0.3	840.7±46.8	400.0±31.0	13.9±0.3	23.4±0.5	811.5±45.9	382.7±34.1
Belarus	12.6±0.4	24.3±0.7	637.1±37.3	286.7±25.9				
Belgium	15.6*±0.3	23.6*±0.6	857.9±51.6	302.0±26.8	14.3±0.4	22.3±0.7	845.7±66.1	285.4±33.5
Bosnia	16.2±0.4	26.9±0.4	1067.1±91.6	339.2±44.2				
Bulgaria	18.1*±0.4	29.4*±0.5	604.1±50.8	217.9±27.2	17.1±0.3	27.8±0.5	572.7±37.3	216.6±25.6
Croatia	18.0±0.4	28.3±0.7	941.2±71.0	324.4±36.6				
Czechia	14.3±0.4	24.4±0.6	636.9±36.2	309.5±25.0				
Denmark	12.6*±0.4	21.1*±0.7	743.6±49.9	262.4±27.3	11.6±0.4	19.8±0.7	710±45.2	235.3±25.0
Estonia	10.7±0.3	22.2±0.7	675.1±40.3	278.3±30.4				
Finland	9.3*±0.3	21.3*±0.7	565.5±32.2	240.0±25.2	8.3±0.5	20.1±0.6	544.1±35.1	224.6±22.7
France	17.2*±0.3	25.2*±0.5	790.4±43.3	244.8±22.2	16.0±0.4	24.0±0.7	769.2±44.9	236.6±25.4
Germany	14.6±0.4	23.6±0.7	697.0±37.3	292.7±21.4				
Greece	20.3*±0.3	30.7*±0.4	680.9±50.1	113.2±13.1	19.7±0.2	29.4±0.5	688.9±50.9	116.7±20.2
Hungary	17.4*±0.4	28.0*±0.7	594.0±50.7	268.1±32.4	16.2±0.3	26.2±0.5	566.7±38.7	257.3±28.4
Ireland	13.8*±0.2	19.0*±0.4	1142.5*±61.0	317.5*±31.2	13.2±0.3	18.6±0.6	1068.8±50.4	280.4±33.5
Italy	18.3*±0.2	27.8*±0.5	823.0±48.5	221.3±25.8	17.3±0.2	26.4±0.4	853.6±49.1	239.4±21.8
Latvia	11.4±0.3	22.6±0.4	678.8±36.9	276.4±26.8				
Lithuania	12.2±0.3	23.3±0.7	676.9±37.7	281.9±29.2				
Moldova	16.3±0.5	28.2±0.6	521.6±34.0	219.5±25.3				
Netherlands	14.8*±0.4	22.6*±0.7	799.5±49.9	282.2±23.5	13.6±0.4	21.3±0.7	769.4±54.2	262±29.6
North Macedonia	17.0±0.3	28.2±0.5	631.3±51.4	208.9±23.4				
Norway	11.0*±0.3	20.5*±0.7	1060.1*±64.8	364.3*±26.8	10.2±0.5	19.5±0.6	980.5±55.8	319.4±35.8
Poland	13.9*±0.4	24.1*±0.6	600.3±33.1	285.5±22.6	12.9±0.4	22.7±0.6	588.8±36.9	282.6±20.9
Portugal	21.7*±0.3	30.1*±0.4	775.6±87.2	102.7±18.7	20.8±0.3	29.3±0.5	782.0±81.8	107.4±25.2
Romania	17.0*±0.4	28.1*±0.6	612.1±44.3	272.8±29.1	15.9±0.3	26.2±0.3	591.1±38.0	281.5±26.1
Russia	12.3±0.4	26.3±0.7	547.5±23.7	218.9±14.6				
Serbia	16.8±0.4	27.9±0.6	832.5±67.3	288.6±34.6				
Slovakia	15.6±0.4	26.2±0.6	664.4±48.6	305.7±32.2				
Slovenia	13.0±0.3	22.2±0.7	1253.9±83.6	498.5±41.1				
Spain	21.2*±0.3	31.4*±0.4	499.4±46.5	103.9±16.5	20.1±0.3	30.2±0.4	524.8±45.0	116.7±16.0
Sweden	11.8*±0.4	21.7*±0.7	635.7*±31.1	256.2*±37.9	10.8±0.5	20.4±0.7	583.6±34.4	217.1±21.3
Switzerland	14.2*±0.3	23.2*±0.7	1183.8±70.6	478.5±39.4	13.1±0.3	22.0±0.6	1171.2±76.3	457.3±34.6
Turkey	18.9*±0.4	30.5*±0.4	610.3±99.2	102.7±18.7	18.1±0.3	29.3±0.4	547.4±34.0	113.2±14.2
Ukraine	14.8±0.4	27.1±0.6	550.4±27.1	227.8±16.5				
United Kingdom	14.0*±0.2	20.6*±0.5	853.4*±50.4	264.6±29.9	13.1±0.3	19.7±0.7	799.8±38.5	244.8±24.6

*Significant difference from 1961–1990 to 1993–2017 ($p < .05$) using Welch t-test. SD = standard deviation.

Table S5 Countrywide averages of wheat harvested areas and total harvests in Europe for 1961 – 1990 and 1993–2017

Country	Harvested area, million ha	Total harvest, million tonnes	Harvested area, million ha	Total harvest, million tonnes
	1993-2017		1961-1990	
Albania	0.1	0.3	0.2	0.4
Austria	0.3	1.5	0.3	1.1
Belarus	0.5	1.4		
Belgium	0.2	1.7	0.2	1.0
Bosnia and Herzegovina	0.1	0.3		
Bulgaria	1.1	3.9	1.1	3.5
Croatia	0.2	0.8		
Czechia	0.8	4.2	1.1	
Denmark	0.6	4.7	0.2	1.1
Estonia	0.1	0.3		
Finland	0.2	0.7	0.2	0.4
France	5.2	35.9	4.4	19.9
Germany	3.0	22.2	2.2	10.3
Greece	0.8	1.9	1.0	2.1
Hungary	1.1	4.5	1.2	4.5
Ireland	0.1	0.7	0.1	0.3
Italy	2.1	7.5	3.6	9.0
Latvia	0.2	0.9		
Lithuania	0.5	1.8		
Montenegro	0.0	0.0		
Netherlands	0.1	1.2	0.1	0.8
North Macedonia	0.1	0.3		
Norway	0.1	0.3	0.0	0.1
Poland	2.4	9.2	1.8	5.2
Portugal	0.1	0.2	0.5	0.5
Moldova	0.4	0.9		
Romania	2.1	6.2	2.5	5.6
Russia	23.4	47.6		
Serbia	0.6	2.3		
Slovakia	0.4	1.7		
Slovenia	0.0	0.2		
Spain	2.1	5.8	3.1	4.8
Sweden	0.4	2.2	0.3	1.3
Switzerland	0.1	0.5	0.1	0.4
Turkey	8.6	19.9	8.8	14.0
Ukraine	6.0	18.7		
United Kingdom	1.9	14.7	1.3	7.2

Table S6 Countrywide averages of maize harvested areas and total harvests in Europe for 1961–1990 and 1993–2017

Country	Harvested area, million ha	Total harvest, million tonnes	Harvested area, million ha	Total harvest, million tonnes
	1993-2017		1961-1990	
Albania	0.1	0.3	0.1	0.3
Austria	0.2	1.9	0.1	1.0
Belarus	0.1	0.5		
Belgium	0.1	0.3		
Bosnia and Herzegovina	0.0	0.8		
Bulgaria	0.4	1.7	0.6	2.3
Croatia	0.3	1.8		
Czechia	0.1	0.5	0.2	0.7
France	1.7	14.8	1.5	8.1
Germany	0.4	3.8	0.1	0.7
Greece	0.2	2.1	0.2	1.0
Hungary	1.1	6.7	1.3	5.5
Italy	1.0	9.0	1.0	5.3
Lithuania	0.0	0.0		
Montenegro	0.0			
Netherlands	0.0	0.2	0.0	0.0
North Macedonia	0.0	0.1		
Poland	0.3	1.9	0.0	0.1
Portugal	0.1	0.8	0.4	0.6
Moldova	0.4	1.3		
Romania	2.7	9.6	3.1	8.4
Russia	1.2	5.0		
Serbia	1.2	5.6		
Slovakia	0.2	0.9		
Slovenia	0.0	0.3		
Spain	0.4	3.9	0.5	2.1
Switzerland	0.0	0.2	0.0	0.1
Turkey	0.6	3.6	0.6	1.3
Ukraine	2.3	11.3		

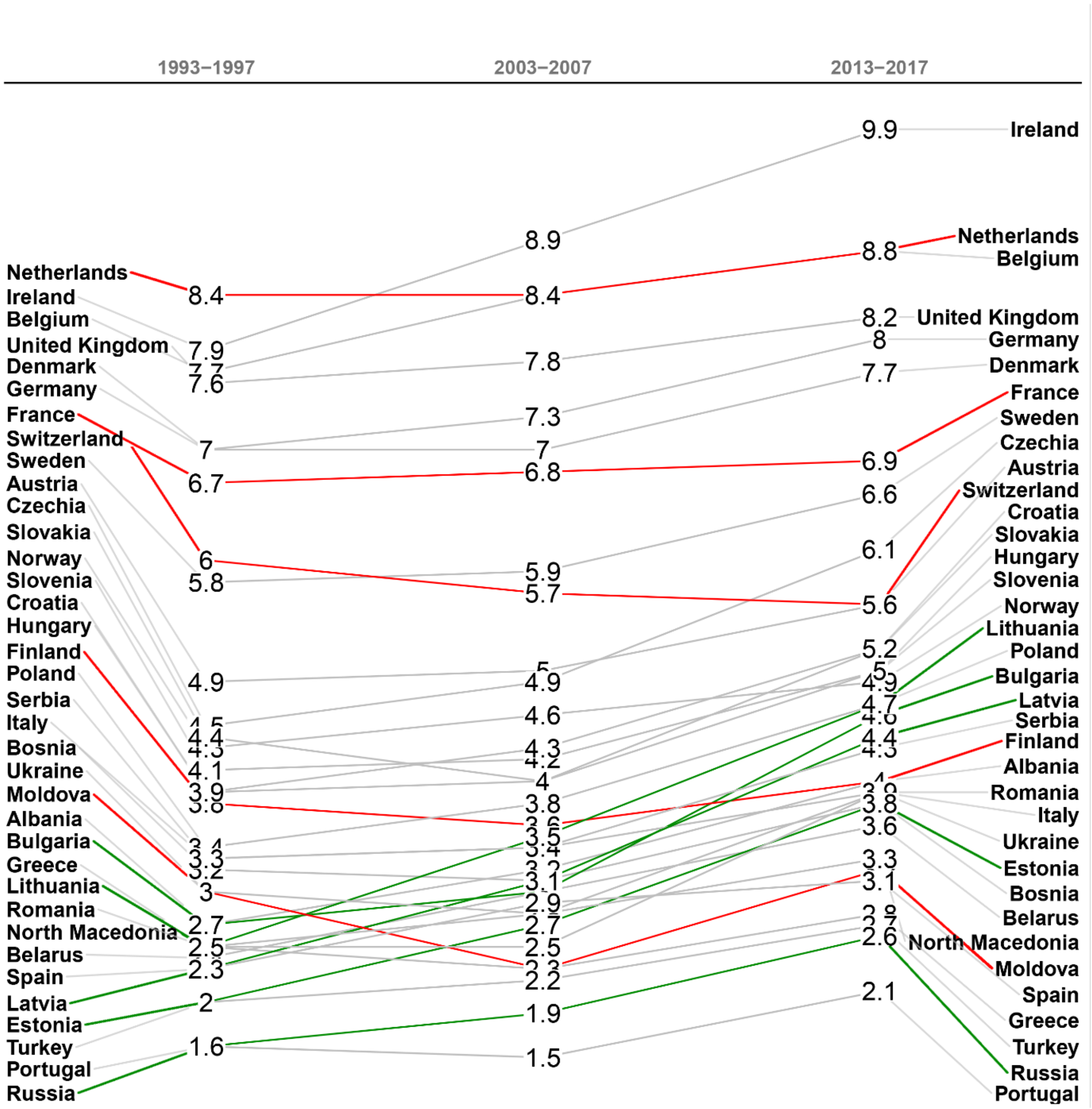

Figure S1 Yield (t ha^{-1}) and rank changes in European wheat yields from the period of 1993–1997 to the period of 2013–2017. Green colour indicates the five countries with the highest yield growth and red one indicates the five lowest yield growth

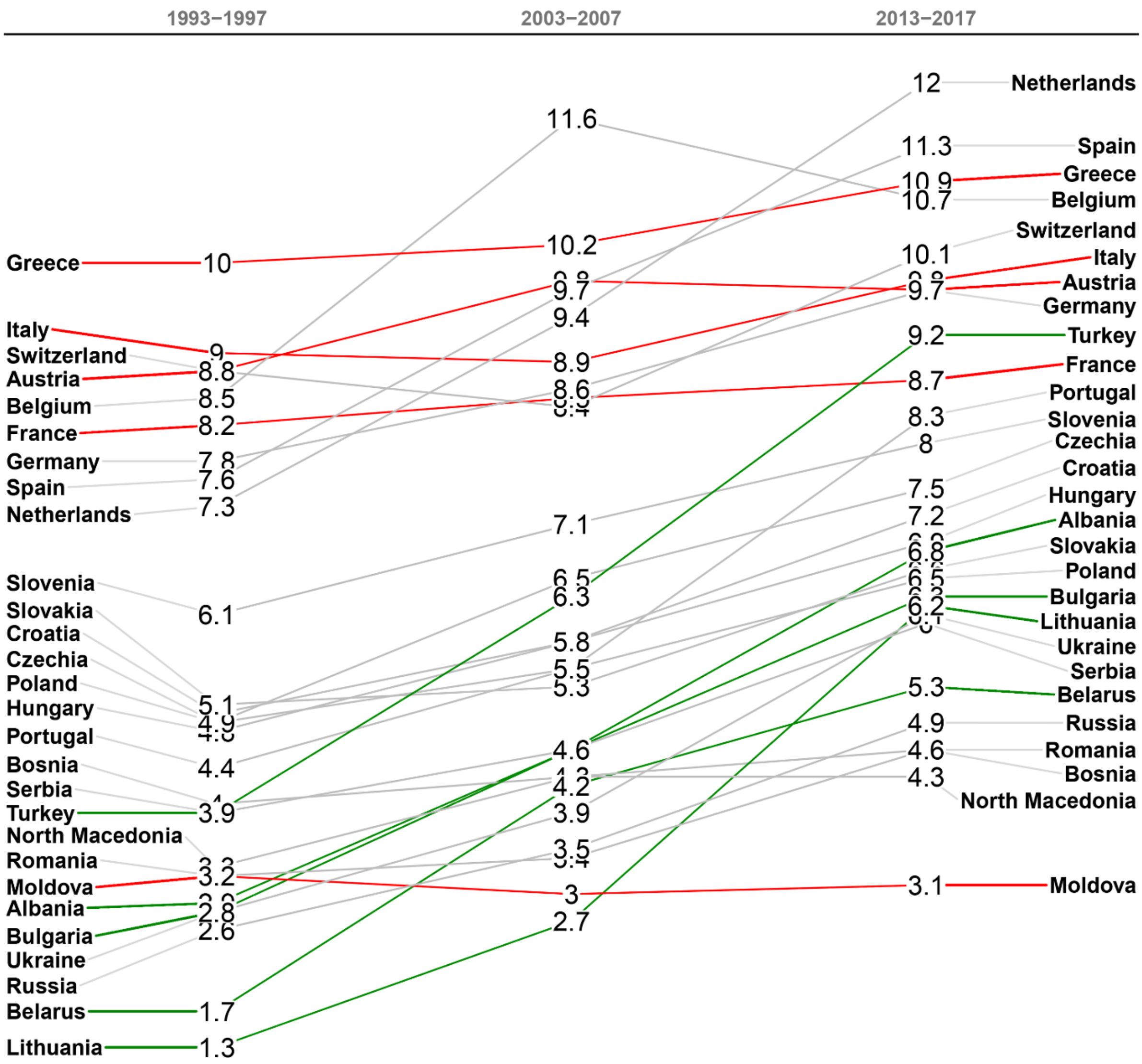

Figure S2 Yield (t ha^{-1}) and rank changes in European maize yields from the period of 1993–1997 to the period of 2013–2017. Green colour indicates the five countries with the highest yield growth and red one indicates the five lowest yield growth.

Table S7 Bootstrap resampling test result for the relationships between GDP per capita and cereal yields in the European countries in 1993–2017

	Wheat		Maize	
	R^2	CI	R^2	CI
Albania	0.86*	0.72–0.92	0.88*	0.95–0.76
Austria	0.11	0.00–0.40	0.28	0.00–0.64
Belarus	0.68*	0.42–0.83	0.70*	0.51–0.80
Belgium	0.24*	0.01–0.51	0.21*	0.02–0.48
Bosnia	0.37*	0.02–0.71	0.07	0.00–0.39
Bulgaria	0.59*	0.19–0.77	0.62*	0.34–0.79
Croatia	0.39*	0.12–0.63	0.30*	0.03–0.60
Czechia	0.45*	0.15–0.65	0.38*	0.08–0.62
Denmark	0.06	0.00–0.36		
Estonia	0.71*	0.53–0.82		
Finland	0.18*	0.01–0.39		
France	<0.01	0.00–0.04	0.18*	0.01–0.45
Germany	0.23*	0.01–0.54	0.42*	0.15–0.66
Greece	0.02	0.00–0.20	0.16	0.00–0.37
Hungary	0.21*	0.02–0.48	0.19*	0.01–0.50
Ireland	0.33*	0.05–0.61		
Italy	0.41*	0.18–0.61	0.01	0.00–0.09
Latvia	0.67*	0.43–0.81		
Lithuania	0.61*	0.29–0.81	0.82*	0.67–0.89
Moldova	0.05	0.00–0.32	0.11	0.00–0.49
Netherlands	0.09	0.00–0.42	0.55*	0.26–0.74
North Macedonia	0.38*	0.09–0.63	0.30*	0.05–0.52
Norway	0.00	0.00–0.00		
Poland	0.70*	0.46–0.63	0.36*	0.05–0.64
Portugal	0.09	0.00–0.33	0.51*	0.27–0.73
Romania	0.31*	0.02–0.60	0.18	0.00–0.51
Russia	0.39*	0.09–0.62	0.58*	0.35–0.73
Serbia	0.37*	0.11–0.59	0.21*	0.01–0.52
Slovakia	0.10	0.00–0.34	0.26*	0.03–0.51
Slovenia	0.22*	0.01–0.50	0.31*	0.04–0.58
Spain	0.26*	0.02–0.51	0.51*	0.30–0.67
Sweden	0.09	0.00–0.37		
Switzerland	0.00	0.00–0.01	0.27*	0.05–0.59
Turkey	0.75*	0.54–0.88	0.89*	0.80–0.94
Ukraine	0.14	0.00–0.39	0.62*	0.34–0.79
United Kingdom	0.07	0.00–0.40		

CI = confidence interval, * = significant association

Table S8 Changing rate of total factor productivity (TFP) and fertiliser use of agriculture (%) in the European countries for 1993–2017 (1993 = 100)

Country	TFP index rate, %	Fertilisers rate, %
Albania	54.0	568
Austria	12.5	74
Belarus	25.3	157
Belgium	-1.5	175
Bosnia and Herzegovina	-2.3	1753
Bulgaria	25.9	225
Croatia	72.2	74
Czechia	-0.7	135
Denmark	20.9	71
Estonia	2.2	151
Finland	0.6	60
France	17.4	64
Germany	23.4	84
Greece	18.1	53
Hungary	19.9	147
Ireland	25.9	80
Italy	19.2	47
Latvia	22.5	235
Lithuania	30.4	252
Netherlands	4.6	53
North Macedonia	43.3	76
Norway	216.7	77
Poland	24.8	130
Portugal	43.2	78
Republic of Moldova	17.4	64
Romania	6.1	124
Russian Federation	29.2	103
Serbia	1.9	231
Slovakia	-1.1	150
Slovenia	-0.5	68
Spain	45.9	92
Sweden	15.3	78
Switzerland	13.0	61
Turkey	34.2	134
Ukraine	62.1	183
United Kingdom	-0.2	68

Source: ERU USDA IAP, FAOSTAT, 2022

Comments on the indices of Table S8

In agricultural economics, TFP and fertiliser changes are treated as better indicators (proxies) behind wheat and maize yield changes than GDP/capita. Therefore, TFP and fertiliser changes were also calculated and results are shown in Table S8. In order to be consistent, changes between 1993-1997 and 2013-2017 were calculated based on TFP indices (source: ERU USDA IAP, 2022) and total fertiliser use (FAOSTAT, 2022). However, results were not found to be reliable due to a number of reasons. First, the two outlier values (Norway, TFP rate, 216.7; Bosnia and Herzegovina, Fertilisers, 1753) is suspected to be a result of a mistake in the original dataset. Second, the value of Estonia seems to be problematic

compared to the other Baltic countries. For both indicators, Estonia performs much worse than Latvia and Lithuania, which is hard to believe given the geographical, historic and economic similarities among these countries. Third, the case of the countries situated in the northern part of Central Europe also seem to be problematic since Czechia and Slovakia show decline in TFP rates, while TFP rates increased in Hungary and Poland. Fourth, the situation in the Balkans also suggests problems – Albania highly outperforms North Macedonia, the Republic of Moldova and Serbia. Fifth, for former Soviet countries, Ukraine seems to have the highest TFP growth, more than double that of Russia and Belarus. Sixth, the highly different performance of the Scandinavian countries also seems unbelievable with the stagnation of Finland's TFP and the soaring of Norway's TFP. Seventh, data for the Western European countries also provides unbelievable results like the comparatively seemingly low productivity increase of Belgium, the Netherlands or the UK. Overall, based on our analysis, GDP/capita turned out to be a better indicator than TFP or fertiliser use in explaining yield changes in our dataset.

Table S9 Coefficients of determination (R^2) between the first-differences of mean monthly and January–March temperature and wheat yields for 1993–2017 and 1961–1990

Country	Jan	Feb	Mar	Apr	May	Jun	Jul	Aug	Sep	Oct	Nov	Dec	Jan–Mar
							1993–2017						
Albania	0.21	0.03	0.02	0.03	0.03	0.01	0.04	0.16	0.02	0.13	0.00	0.18	0.03
Austria	0.05	0.19	0.00	0.00	0.45	0.24	0.00	0.12	0.14	0.01	0.03	0.04	0.12
Belarus	0.14	0.10	0.29	0.00	0.09	0.19	0.13	0.03	0.00	0.00	0.03	0.00	0.38
Belgium	0.07	0.12	0.00	0.00	0.08	0.00	0.01	0.00	0.19	0.00	0.04	0.07	0.11
Bosnia	0.04	0.17	0.03	0.23	0.05	0.01	0.00	0.00	0.04	0.13	0.00	0.07	0.01
Bulgaria	0.18	0.04	0.19	0.07	0.49	0.13	0.05	0.13	0.00	0.01	0.01	0.03	0.01
Croatia	0.03	0.14	0.01	0.06	0.07	0.01	0.01	0.00	0.01	0.25	0.00	0.08	0.01
Czech	0.03	0.17	0.00	0.02	0.36	0.16	0.03	0.06	0.05	0.08	0.04	0.03	0.09
Denmark	0.04	0.13	0.02	0.01	0.00	0.16	0.04	0.01	0.02	0.08	0.03	0.01	0.09
Estonia	0.37	0.00	0.13	0.02	0.04	0.18	0.26	0.04	0.08	0.01	0.04	0.04	0.21
Finland	0.08	0.00	0.02	0.08	0.00	0.15	0.03	0.10	0.04	0.00	0.21	0.05	0.02
France	0.02	0.02	0.00	0.00	0.02	0.01	0.01	0.01	0.07	0.03	0.02	0.11	0.03
Germany	0.00	0.02	0.00	0.01	0.19	0.51	0.00	0.00	0.00	0.29	0.00	0.01	0.00
Greece	0.01	0.17	0.12	0.28	0.13	0.06	0.00	0.03	0.09	0.00	0.01	0.03	0.07
Hungary	0.02	0.26	0.07	0.00	0.52	0.15	0.01	0.04	0.06	0.04	0.02	0.01	0.20
Ireland	0.08	0.05	0.22	0.04	0.00	0.31	0.05	0.12	0.06	0.04	0.15	0.06	0.16
Italy	0.04	0.02	0.10	0.11	0.24	0.01	0.01	0.13	0.00	0.09	0.01	0.03	0.01
Latvia	0.33	0.01	0.06	0.00	0.00	0.07	0.22	0.03	0.01	0.12	0.01	0.13	0.19
Lithuania	0.26	0.04	0.23	0.00	0.00	0.10	0.34	0.08	0.06	0.04	0.00	0.04	0.32
Macedonia	0.00	0.34	0.08	0.30	0.57	0.02	0.05	0.20	0.01	0.00	0.03	0.03	0.14
Moldova	0.09	0.09	0.02	0.04	0.17	0.04	0.12	0.21	0.20	0.09	0.17	0.02	0.01
Netherlands	0.14	0.15	0.04	0.03	0.06	0.06	0.00	0.01	0.05	0.06	0.00	0.03	0.20
Norway	0.08	0.27	0.03	0.03	0.04	0.03	0.19	0.09	0.09	0.05	0.14	0.19	0.19
Poland	0.04	0.19	0.12	0.05	0.00	0.02	0.01	0.01	0.05	0.04	0.19	0.26	0.22
Portugal	0.09	0.01	0.02	0.04	0.16	0.07	0.02	0.24	0.01	0.00	0.04	0.09	0.03
Romania	0.04	0.07	0.15	0.01	0.54	0.26	0.01	0.17	0.00	0.02	0.17	0.06	0.04
Russia	0.00	0.08	0.12	0.04	0.18	0.18	0.01	0.13	0.04	0.00	0.04	0.00	0.11
Serbia	0.01	0.28	0.11	0.01	0.51	0.15	0.00	0.18	0.02	0.10	0.16	0.00	0.21
Slovakia	0.06	0.25	0.04	0.00	0.46	0.17	0.00	0.07	0.15	0.02	0.08	0.04	0.21
Slovenia	0.08	0.21	0.07	0.00	0.22	0.02	0.00	0.08	0.01	0.07	0.01	0.00	0.25
Spain	0.19	0.20	0.00	0.02	0.37	0.11	0.01	0.10	0.04	0.20	0.02	0.01	0.23
Sweden	0.38	0.09	0.36	0.04	0.05	0.14	0.13	0.01	0.07	0.02	0.00	0.14	0.43
Switzerland	0.01	0.07	0.00	0.09	0.00	0.15	0.15	0.04	0.13	0.09	0.00	0.12	0.02
Turkey	0.19	0.03	0.10	0.00	0.02	0.09	0.14	0.11	0.02	0.17	0.01	0.08	0.16
Ukraine	0.00	0.12	0.08	0.00	0.22	0.12	0.00	0.16	0.02	0.01	0.28	0.00	0.10
UK	0.01	0.01	0.01	0.03	0.01	0.07	0.01	0.03	0.02	0.00	0.04	0.04	0.00
							1961–1990						
Albania	0.06	0.00	0.07	0.05	0.01	0.07	0.00	0.00	0.12	0.01	0.01	0.00	0.0
Austria	0.01	0.02	0.07	0.02	0.02	0.28	0.00	0.00	0.02	0.01	0.07	0.00	0.0
Belgium	0.06	0.03	0.00	0.17	0.06	0.01	0.03	0.12	0.09	0.00	0.06	0.01	0.0
Bulgaria	0.03	0.02	0.00	0.01	0.27	0.02	0.06	0.01	0.08	0.20	0.00	0.03	0.0
Denmark	0.17	0.22	0.06	0.03	0.01	0.00	0.15	0.01	0.00	0.09	0.11	0.10	0.3
Finland	0.04	0.02	0.09	0.15	0.00	0.09	0.01	0.04	0.00	0.01	0.00	0.01	0.1
France	0.05	0.02	0.00	0.06	0.02	0.29	0.19	0.04	0.12	0.13	0.00	0.00	0.1
Greece	0.02	0.00	0.01	0.02	0.32	0.10	0.23	0.01	0.01	0.10	0.21	0.03	0.0
Hungary	0.01	0.01	0.03	0.11	0.08	0.40	0.04	0.01	0.00	0.02	0.12	0.00	0.0
Ireland	0.04	0.07	0.01	0.23	0.17	0.02	0.09	0.04	0.00	0.04	0.03	0.06	0.0

Italy	0.03	0.05	0.18	0.06	0.06	0.07	0.13	0.01	0.00	0.02	0.01	0.00	0.1
Netherlands	0.24	0.00	0.02	0.36	0.02	0.00	0.06	0.01	0.06	0.05	0.15	0.01	0.1
Norway	0.04	0.19	0.01	0.21	0.00	0.04	0.14	0.00	0.04	0.06	0.02	0.01	0.0
Poland	0.01	0.00	0.00	0.00	0.03	0.15	0.17	0.09	0.01	0.06	0.08	0.07	0.0
Portugal	0.15	0.06	0.02	0.01	0.01	0.00	0.00	0.02	0.14	0.07	0.02	0.07	0.3
Romania	0.01	0.00	0.03	0.17	0.05	0.17	0.05	0.06	0.05	0.01	0.03	0.00	0.0
Spain	0.00	0.04	0.13	0.24	0.15	0.19	0.06	0.01	0.00	0.01	0.07	0.05	0.0
Sweden	0.14	0.33	0.19	0.30	0.04	0.03	0.05	0.00	0.00	0.05	0.18	0.01	0.4
Switzerland	0.09	0.01	0.00	0.02	0.00	0.03	0.00	0.01	0.24	0.06	0.01	0.02	0.1
Turkey	0.13	0.01	0.05	0.05	0.01	0.01	0.00	0.00	0.08	0.00	0.04	0.01	0.0
UK	0.06	0.04	0.01	0.22	0.01	0.02	0.11	0.00	0.10	0.02	0.09	0.03	0.0

Table S10 Coefficients of determination (R^2) between the first-differences of mean monthly and May–July PDSI and wheat yields for 1993–2017 and 1961–1990

Country	Jan	Feb	Mar	Apr	May	Jun	Jul	Aug	Sep	Oct	Nov	Dec	May–Jul
							1993–2017						
Albania	0.27	0.37	0.23	0.24	0.20	0.16	0.06	0.05	0.05	0.10	0.05	0.01	0.01
Austria	0.00	0.01	0.04	0.12	0.12	0.25	0.26	0.18	0.04	0.02	0.01	0.00	0.23
Belarus	0.01	0.03	0.04	0.01	0.01	0.00	0.00	0.01	0.02	0.06	0.08	0.06	0.00
Belgium	0.11	0.17	0.15	0.15	0.18	0.36	0.21	0.11	0.05	0.04	0.01	0.00	0.28
Bosnia	0.11	0.07	0.03	0.02	0.01	0.02	0.03	0.03	0.06	0.14	0.13	0.14	0.02
Bulgaria	0.00	0.00	0.00	0.00	0.00	0.03	0.07	0.06	0.01	0.00	0.02	0.04	0.03
Croatia	0.14	0.17	0.08	0.04	0.05	0.06	0.08	0.07	0.10	0.10	0.09	0.09	0.06
Czech	0.02	0.04	0.00	0.00	0.01	0.02	0.06	0.03	0.02	0.01	0.00	0.00	0.03
Denmark	0.02	0.03	0.01	0.00	0.01	0.04	0.09	0.09	0.08	0.02	0.00	0.00	0.05
Estonia	0.02	0.00	0.05	0.04	0.09	0.08	0.13	0.03	0.07	0.01	0.05	0.03	0.12
Finland	0.09	0.15	0.14	0.10	0.04	0.00	0.02	0.01	0.01	0.00	0.02	0.01	0.00
France	0.10	0.06	0.06	0.02	0.04	0.12	0.06	0.01	0.01	0.02	0.02	0.04	0.07
Germany	0.12	0.01	0.00	0.00	0.01	0.01	0.01	0.00	0.00	0.00	0.00	0.00	0.01
Greece	0.04	0.05	0.04	0.01	0.05	0.02	0.00	0.00	0.00	0.00	0.04	0.03	0.02
Hungary	0.00	0.00	0.02	0.08	0.09	0.14	0.14	0.10	0.07	0.01	0.00	0.00	0.13
Ireland	0.04	0.02	0.00	0.10	0.13	0.36	0.47	0.30	0.15	0.02	0.02	0.01	0.33
Italy	0.29	0.08	0.06	0.00	0.05	0.03	0.07	0.08	0.20	0.23	0.17	0.14	0.05
Latvia	0.07	0.05	0.09	0.08	0.07	0.03	0.04	0.01	0.00	0.00	0.00	0.03	0.05
Lithuania	0.00	0.00	0.01	0.00	0.00	0.05	0.10	0.02	0.01	0.01	0.02	0.03	0.05
Macedonia	0.04	0.07	0.00	0.01	0.02	0.06	0.33	0.19	0.13	0.08	0.00	0.01	0.14
Moldova	0.00	0.03	0.00	0.03	0.18	0.30	0.38	0.35	0.34	0.29	0.23	0.03	0.30
Netherlands	0.02	0.08	0.08	0.12	0.15	0.30	0.30	0.15	0.09	0.09	0.04	0.07	0.28
Norway	0.00	0.01	0.00	0.00	0.00	0.00	0.02	0.00	0.00	0.00	0.00	0.03	0.00
Poland	0.03	0.09	0.11	0.05	0.11	0.22	0.27	0.16	0.15	0.12	0.10	0.09	0.24
Portugal	0.14	0.09	0.05	0.00	0.01	0.02	0.02	0.13	0.22	0.21	0.14	0.13	0.02
Romania	0.01	0.00	0.00	0.03	0.08	0.20	0.31	0.19	0.09	0.01	0.01	0.00	0.20
Russia	0.00	0.00	0.00	0.01	0.04	0.10	0.09	0.06	0.18	0.20	0.04	0.02	0.09
Serbia	0.05	0.03	0.01	0.00	0.01	0.05	0.08	0.08	0.02	0.01	0.01	0.04	0.04
Slovakia	0.03	0.02	0.00	0.00	0.01	0.04	0.10	0.09	0.03	0.03	0.01	0.00	0.05
Slovenia	0.30	0.26	0.24	0.12	0.12	0.02	0.03	0.07	0.24	0.30	0.17	0.14	0.02
Spain	0.11	0.16	0.23	0.46	0.57	0.50	0.42	0.38	0.29	0.19	0.11	0.02	0.51
Sweden	0.06	0.05	0.05	0.02	0.04	0.11	0.28	0.02	0.02	0.00	0.00	0.00	0.15
Switzerland	0.16	0.16	0.25	0.33	0.39	0.53	0.23	0.10	0.06	0.06	0.01	0.00	0.44
Turkey	0.19	0.20	0.22	0.32	0.38	0.34	0.28	0.18	0.09	0.04	0.02	0.10	0.33

Ukraine	0.00	0.04	0.02	0.01	0.04	0.10	0.13	0.07	0.05	0.03	0.01	0.00	0.09
UK	0.00	0.00	0.01	0.02	0.02	0.19	0.17	0.09	0.09	0.05	0.02	0.02	0.12
	1961–1990												
Albania	0.08	0.05	0.00	0.00	0.02	0.00	0.01	0.01	0.00	0.00	0.09	0.11	0.01
Austria	0.01	0.06	0.12	0.24	0.17	0.28	0.35	0.29	0.21	0.08	0.06	0.04	0.30
Belgium	0.00	0.03	0.03	0.08	0.05	0.12	0.08	0.09	0.02	0.00	0.01	0.00	0.09
Bulgaria	0.07	0.10	0.15	0.18	0.12	0.04	0.05	0.07	0.03	0.04	0.02	0.02	0.07
Denmark	0.04	0.02	0.00	0.02	0.03	0.00	0.00	0.00	0.00	0.00	0.00	0.00	0.01
Finland	0.00	0.03	0.06	0.06	0.02	0.19	0.29	0.25	0.36	0.25	0.18	0.11	0.18
France	0.01	0.00	0.00	0.02	0.00	0.00	0.00	0.03	0.01	0.03	0.01	0.00	0.00
Greece	0.01	0.03	0.10	0.22	0.22	0.19	0.17	0.08	0.19	0.14	0.05	0.00	0.21
Hungary	0.13	0.12	0.13	0.12	0.02	0.02	0.02	0.04	0.01	0.02	0.02	0.01	0.02
Ireland	0.01	0.04	0.00	0.01	0.09	0.03	0.06	0.07	0.00	0.00	0.00	0.01	0.06
Italy	0.20	0.16	0.11	0.05	0.00	0.00	0.01	0.03	0.00	0.01	0.07	0.09	0.00
Netherlands	0.04	0.01	0.01	0.03	0.03	0.08	0.14	0.21	0.10	0.04	0.09	0.03	0.08
Norway	0.01	0.01	0.05	0.17	0.20	0.03	0.04	0.05	0.01	0.14	0.23	0.15	0.09
Poland	0.00	0.00	0.02	0.09	0.03	0.03	0.09	0.08	0.03	0.00	0.00	0.00	0.06
Portugal	0.31	0.24	0.12	0.10	0.08	0.08	0.03	0.00	0.00	0.00	0.02	0.03	0.06
Romania	0.06	0.10	0.11	0.13	0.08	0.05	0.01	0.02	0.00	0.00	0.01	0.00	0.05
Spain	0.00	0.00	0.03	0.05	0.16	0.17	0.18	0.10	0.03	0.01	0.06	0.04	0.18
Sweden	0.00	0.02	0.05	0.16	0.19	0.24	0.18	0.18	0.10	0.03	0.03	0.01	0.22
Switzerland	0.00	0.01	0.01	0.05	0.06	0.23	0.31	0.30	0.07	0.09	0.19	0.05	0.21
Turkey	0.09	0.12	0.16	0.33	0.48	0.50	0.50	0.51	0.46	0.30	0.17	0.08	0.51
UK	0.00	0.01	0.01	0.01	0.01	0.01	0.03	0.01	0.11	0.13	0.03	0.03	0.02

Table S11 Coefficients of determination (R^2) between the first-differences of mean monthly and July–August PDSI and maize yields for 1993–2017 and 1961–1990

Country	Apr	May	Jun	Jul	Aug	Sep	Oct	Jul–Aug
	1993–2017							
Albania	0.22	0.21	0.18	0.14	0.12	0.10	0.09	0.13
Austria	0.07	0.11	0.07	0.03	0.04	0.01	0.02	0.04
Belarus	0.07	0.06	0.06	0.00	0.00	0.00	0.01	0.00
Belgium	0.04	0.05	0.02	0.01	0.00	0.00	0.00	0.00
Bosnia	0.46	0.47	0.58	0.66	0.65	0.52	0.38	0.66
Bulgaria	0.00	0.00	0.03	0.17	0.27	0.15	0.18	0.22
Croatia	0.38	0.39	0.52	0.65	0.61	0.41	0.30	0.63
Czech	0.04	0.05	0.02	0.07	0.04	0.00	0.03	0.06
France	0.07	0.03	0.01	0.01	0.12	0.00	0.00	0.05
Germany	0.03	0.07	0.02	0.17	0.10	0.04	0.05	0.14
Greece	0.10	0.04	0.03	0.00	0.01	0.00	0.00	0.00
Hungary	0.14	0.20	0.30	0.46	0.42	0.30	0.23	0.44
Italy	0.01	0.06	0.15	0.30	0.29	0.18	0.12	0.30
Lithuania	0.24	0.27	0.18	0.33	0.21	0.15	0.10	0.29
Macedonia	0.04	0.08	0.17	0.55	0.54	0.43	0.39	0.57
Moldova	0.01	0.11	0.34	0.26	0.31	0.30	0.29	0.29
Netherlands	0.03	0.05	0.04	0.03	0.02	0.04	0.10	0.02
Poland	0.04	0.05	0.00	0.18	0.02	0.00	0.06	0.09
Portugal	0.01	0.01	0.00	0.00	0.01	0.01	0.00	0.00
Romania	0.09	0.14	0.25	0.40	0.35	0.22	0.18	0.38
Russia	0.00	0.01	0.03	0.14	0.19	0.47	0.44	0.17

Serbia	0.26	0.37	0.53	0.69	0.70	0.54	0.41	0.70
Slovakia	0.00	0.00	0.01	0.14	0.16	0.05	0.06	0.15
Slovenia	0.04	0.01	0.01	0.10	0.24	0.22	0.20	0.18
Spain	0.25	0.27	0.23	0.22	0.18	0.17	0.06	0.20
Switzerland	0.00	0.01	0.00	0.06	0.10	0.06	0.05	0.08
Turkey	0.00	0.00	0.00	0.00	0.00	0.00	0.00	0.00
Ukraine	0.00	0.00	0.05	0.07	0.04	0.05	0.02	0.06
				1961–1990				
Albania	0.06	0.04	0.00	0.03	0.03	0.05	0.03	0.03
Austria	0.00	0.07	0.00	0.01	0.01	0.06	0.06	0.00
Belgium	0.02	0.01	0.00	0.03	0.03	0.10	0.06	0.04
Bulgaria	0.02	0.00	0.02	0.08	0.21	0.18	0.24	0.12
France	0.01	0.02	0.14	0.22	0.21	0.27	0.20	0.28
Greece	0.02	0.01	0.01	0.05	0.09	0.05	0.02	0.04
Hungary	0.07	0.07	0.15	0.21	0.28	0.03	0.01	0.23
Italy	0.02	0.00	0.01	0.10	0.10	0.08	0.05	0.08
Netherlands	0.00	0.01	0.00	0.00	0.00	0.01	0.03	0.00
Poland	0.19	0.08	0.02	0.00	0.00	0.06	0.05	0.00
Portugal	0.01	0.01	0.00	0.02	0.01	0.05	0.08	0.01
Romania	0.48	0.40	0.28	0.09	0.01	0.05	0.11	0.01
Spain	0.01	0.03	0.04	0.06	0.02	0.00	0.00	0.05
Switzerland	0.02	0.01	0.01	0.00	0.07	0.00	0.02	0.01
Turkey	0.03	0.00	0.00	0.01	0.00	0.02	0.01	0.01

Table S12 Coefficients of determination (R^2) between the first-differences of mean monthly and May–July SPEI1 and wheat yields for 1993–2017 and 1961–1990

Country	Jan	Feb	Mar	Apr	May	Jun	Jul	Aug	Sep	Oct	Nov	Dec	May–Jul
							1993–2017						
Albania	0.24	0.46	0.01	0.02	0.00	0.03	0.01	0.00	0.05	0.07	0.07	0.03	0.04
Austria	0.13	0.03	0.08	0.07	0.04	0.29	0.00	0.01	0.05	0.05	0.03	0.07	0.30
Belarus	0.11	0.03	0.02	0.02	0.01	0.05	0.00	0.02	0.00	0.00	0.12	0.05	0.02
Belgium	0.09	0.09	0.04	0.10	0.01	0.44	0.01	0.11	0.04	0.01	0.20	0.01	0.26
Bosnia	0.09	0.07	0.08	0.00	0.01	0.01	0.03	0.00	0.17	0.00	0.00	0.03	0.01
Bulgaria	0.02	0.23	0.01	0.08	0.04	0.30	0.13	0.00	0.04	0.16	0.13	0.04	0.14
Croatia	0.17	0.26	0.08	0.00	0.02	0.01	0.03	0.02	0.09	0.01	0.01	0.02	0.04
Czech	0.09	0.00	0.10	0.02	0.03	0.05	0.01	0.01	0.00	0.01	0.01	0.05	0.12
Denmark	-0.75	-0.39	0.46	0.19	0.78	1.11	0.81	1.26	3.05	1.80	0.56	1.22	0.90
Estonia	0.06	0.04	0.00	0.00	0.11	0.03	0.09	0.06	0.11	0.01	0.01	0.00	0.18
Finland	0.00	0.21	0.06	0.01	0.05	0.05	0.10	0.00	0.01	0.01	0.15	0.00	0.14
France	0.04	0.07	0.02	0.01	0.04	0.30	0.00	0.15	0.15	0.07	0.01	0.04	0.18
Germany	0.02	0.00	0.05	0.11	0.02	0.00	0.00	0.02	0.00	0.00	0.00	0.00	0.00
Greece	0.01	0.09	0.01	0.01	0.02	0.01	0.01	0.00	0.00	0.01	0.04	0.01	0.00
Hungary	0.00	0.05	0.18	0.21	0.01	0.20	0.02	0.00	0.00	0.14	0.01	0.04	0.16
Ireland	0.01	0.02	0.06	0.01	0.04	0.17	0.05	0.00	0.00	0.08	0.08	0.02	0.04
Italy	0.15	0.00	0.05	0.01	0.22	0.02	0.00	0.06	0.23	0.07	0.01	0.00	0.15
Latvia	0.15	0.02	0.02	0.00	0.01	0.20	0.12	0.01	0.00	0.03	0.07	0.03	0.23
Lithuania	0.13	0.01	0.02	0.00	0.00	0.15	0.13	0.00	0.01	0.03	0.15	0.05	0.17
Macedonia	0.00	0.24	0.06	0.09	0.09	0.27	0.44	0.01	0.05	0.13	0.05	0.06	0.67
Moldova	0.01	0.43	0.03	0.00	0.01	0.02	0.29	0.00	0.01	0.34	0.00	0.04	0.10
Netherlands	0.08	0.07	0.03	0.01	0.12	0.00	0.02	0.01	0.04	0.00	0.02	0.06	0.01

Norway	0.24	0.00	0.04	0.08	0.00	0.15	0.01	0.03	0.11	0.02	0.21	0.00	0.04
Poland	0.05	0.03	0.06	0.01	0.28	0.01	0.09	0.00	0.10	0.05	0.24	0.28	0.29
Portugal	0.00	0.01	0.01	0.13	0.10	0.15	0.23	0.18	0.11	0.05	0.02	0.02	0.17
Romania	0.02	0.04	0.01	0.00	0.01	0.00	0.01	0.01	0.01	0.13	0.16	0.07	0.00
Russia	0.03	0.07	0.06	0.01	0.03	0.01	0.00	0.00	0.02	0.07	0.07	0.03	0.01
Serbia	0.22	0.20	0.10	0.04	0.00	0.01	0.05	0.02	0.00	0.04	0.09	0.07	0.01
Slovakia	0.02	0.02	0.00	0.02	0.03	0.07	0.13	0.11	0.05	0.01	0.00	0.00	0.07
Slovenia	0.42	0.29	0.29	0.11	0.02	0.01	0.00	0.00	0.02	0.00	0.00	0.01	0.00
Spain	0.02	0.02	0.01	0.00	0.01	0.02	0.01	0.00	0.00	0.01	0.01	0.03	0.01
Sweden	0.06	0.03	0.02	0.01	0.02	0.03	0.02	0.00	0.00	0.00	0.01	0.15	0.02
Switzerland	0.01	0.00	0.02	0.02	0.04	0.05	0.03	0.03	0.01	0.02	0.00	0.03	0.04
Turkey	0.00	0.00	0.00	0.05	0.09	0.09	0.11	0.09	0.07	0.12	0.07	0.07	0.10
Ukraine	0.06	0.15	0.07	0.03	0.02	0.00	0.00	0.00	0.00	0.03	0.06	0.01	0.00
UK	0.16	0.14	0.16	0.21	0.07	0.04	0.05	0.10	0.11	0.08	0.08	0.16	0.05
							1961–1990						
Albania	0.02	0.00	0.09	0.03	0.08	0.00	0.02	0.04	0.10	0.03	0.21	0.07	0.04
Austria	0.01	0.18	0.04	0.11	0.00	0.08	0.21	0.04	0.01	0.11	0.00	0.07	0.14
Belgium	0.03	0.06	0.03	0.15	0.01	0.15	0.05	0.03	0.27	0.10	0.10	0.14	0.11
Bulgaria	0.00	0.02	0.13	0.15	0.00	0.12	0.00	0.01	0.03	0.03	0.05	0.01	0.04
Denmark	0.22	0.00	0.03	0.20	0.03	0.00	0.00	0.03	0.00	0.02	0.06	0.00	0.00
Finland	0.00	0.13	0.05	0.00	0.01	0.26	0.09	0.01	0.12	0.00	0.02	0.02	0.24
France	0.06	0.01	0.05	0.22	0.04	0.01	0.00	0.04	0.10	0.03	0.00	0.02	0.00
Greece	0.00	0.02	0.17	0.16	0.09	0.00	0.01	0.00	0.05	0.04	0.05	0.11	0.04
Hungary	0.03	0.00	0.06	0.00	0.08	0.01	0.00	0.04	0.14	0.03	0.01	0.00	0.04
Ireland	0.10	0.11	0.14	0.01	0.16	0.00	0.04	0.03	0.01	0.04	0.03	0.05	0.18
Italy	0.00	0.00	0.02	0.05	0.10	0.05	0.08	0.07	0.01	0.02	0.13	0.00	0.01
Netherlands	0.04	0.01	0.03	0.21	0.02	0.15	0.06	0.18	0.11	0.22	0.35	0.18	0.20
Norway	0.00	0.00	0.04	0.24	0.03	0.24	0.01	0.08	0.08	0.15	0.08	0.01	0.06
Poland	0.03	0.02	0.13	0.26	0.05	0.00	0.04	0.05	0.05	0.10	0.01	0.04	0.00
Portugal	0.07	0.10	0.09	0.01	0.00	0.08	0.01	0.11	0.12	0.00	0.09	0.01	0.03
Romania	0.01	0.06	0.14	0.13	0.00	0.01	0.01	0.07	0.06	0.05	0.01	0.10	0.00
Spain	0.03	0.00	0.07	0.00	0.47	0.12	0.01	0.14	0.03	0.01	0.20	0.07	0.27
Sweden	0.00	0.10	0.06	0.29	0.05	0.00	0.01	0.02	0.01	0.13	0.10	0.01	0.04
Switzerland	0.00	0.02	0.02	0.19	0.00	0.30	0.12	0.18	0.09	0.00	0.08	0.07	0.32
Turkey	0.02	0.01	0.02	0.38	0.22	0.06	0.14	0.01	0.00	0.00	0.10	0.01	0.20
UK	0.20	0.05	0.01	0.18	0.04	0.01	0.05	0.05	0.00	0.02	0.14	0.01	0.15

Table S13 Coefficients of determination (R^2) between the first-differences of mean monthly and July-August and May-August SPEI1 and maize yields for 1993–2017 and 1961–1990

Country	Apr	May	Jun	Jul	Aug	Sep	Oct	Jul–Aug
				1993–2017				
Albania	0.00	0.00	0.05	0.00	0.00	0.09	0.03	0.01
Austria	0.00	0.18	0.00	0.74	0.06	0.03	0.01	0.54
Belarus	0.07	0.00	0.04	0.23	0.00	0.10	0.00	0.18
Belgium	0.24	0.06	0.01	0.07	0.01	0.02	0.14	0.07
Bosnia	0.14	0.20	0.12	0.55	0.16	0.38	0.00	0.41
Bulgaria	0.01	0.00	0.65	0.48	0.31	0.01	0.02	0.59
Croatia	0.29	0.07	0.47	0.21	0.34	0.00	0.15	0.43
Czech	0.02	0.02	0.02	0.35	0.04	0.07	0.05	0.32
France	0.09	0.00	0.01	0.47	0.48	0.18	0.03	0.85

Germany	0.03	0.03	0.06	0.69	0.02	0.03	0.00	0.55
Greece	0.18	0.25	0.03	0.01	0.22	0.03	0.00	0.13
Hungary	0.19	0.03	0.38	0.41	0.19	0.01	0.14	0.50
Italy	0.05	0.09	0.08	0.24	0.02	0.03	0.07	0.16
Lithuania	0.04	0.27	0.01	0.02	0.01	0.00	0.01	0.00
Macedonia	0.02	0.01	0.17	0.26	0.32	0.08	0.01	0.43
Moldova	0.02	0.02	0.02	0.17	0.02	0.00	0.07	0.03
Netherlands	0.03	0.10	0.14	0.05	0.13	0.09	0.00	0.13
Norway	0.00	0.30	0.11	0.00	0.01	0.00	0.02	0.00
Poland	0.00	0.30	0.11	0.00	0.01	0.00	0.02	0.00
Portugal	0.05	0.08	0.05	0.04	0.04	0.10	0.16	0.04
Romania	0.06	0.03	0.05	0.11	0.09	0.01	0.00	0.10
Russia	0.00	0.00	0.00	0.00	0.01	0.00	0.03	0.01
Serbia	0.22	0.32	0.40	0.52	0.48	0.34	0.20	0.51
Slovakia	0.06	0.14	0.19	0.26	0.22	0.23	0.12	0.25
Slovenia	0.04	0.10	0.11	0.21	0.23	0.26	0.28	0.22
Spain	0.01	0.02	0.03	0.04	0.02	0.01	0.00	0.03
Switzerland	0.06	0.02	0.00	0.01	0.00	0.00	0.01	0.00
Turkey	0.05	0.03	0.02	0.01	0.01	0.02	0.01	0.01
	1961–1990							
Albania	0.00	0.00	0.22	0.38	0.02	0.01	0.00	0.3
Austria	0.12	0.45	0.24	0.04	0.03	0.07	0.04	0.0
Belgium	0.01	0.02	0.03	0.15	0.16	0.06	0.00	0.2
Bulgaria	0.00	0.07	0.04	0.48	0.08	0.00	0.13	0.4
France	0.00	0.02	0.29	0.24	0.02	0.01	0.03	0.4
Greece	0.02	0.00	0.04	0.12	0.02	0.01	0.01	0.1
Hungary	0.02	0.01	0.21	0.17	0.27	0.38	0.05	0.3
Italy	0.02	0.02	0.11	0.31	0.01	0.05	0.00	0.2
Netherlands	0.10	0.13	0.03	0.01	0.13	0.12	0.15	0.0
Poland	0.02	0.19	0.04	0.01	0.09	0.25	0.01	0.1
Portugal	0.03	0.03	0.01	0.17	0.00	0.01	0.07	0.1
Romania	0.01	0.33	0.00	0.09	0.05	0.00	0.05	0.2
Spain	0.09	0.03	0.08	0.11	0.15	0.02	0.00	0.0
Switzerland	0.04	0.05	0.00	0.04	0.13	0.02	0.00	0.0
Turkey	0.01	0.09	0.11	0.00	0.00	0.04	0.00	0.0

Table S14 Coefficients of determination (R^2) between the first-differences of mean monthly and May–July SPEI3 and wheat yields for 1993–2017 and 1961–1990

Country	Jan	Feb	Mar	Apr	May	Jun	Jul	Aug	Sep	Oct	Nov	Dec	May–Jul
	1993–2017												
Albania	0.03	0.00	0.01	0.00	0.01	0.00	0.01	0.01	0.00	0.01	0.03	0.04	0.11
Austria	0.00	0.00	0.04	0.07	0.16	0.30	0.16	0.15	0.00	0.00	0.00	0.02	0.26
Belarus	0.00	0.00	0.13	0.02	0.13	0.02	0.06	0.00	0.01	0.00	0.00	0.02	0.09
Belgium	0.03	0.04	0.05	0.11	0.12	0.08	0.00	0.03	0.00	0.04	0.01	0.01	0.07
Bosnia	0.15	0.17	0.07	0.06	0.00	0.00	0.04	0.15	0.04	0.00	0.06	0.10	0.02
Bulgaria	0.01	0.14	0.03	0.19	0.22	0.11	0.01	0.02	0.01	0.03	0.00	0.04	0.11
Croatia	0.04	0.09	0.07	0.11	0.01	0.00	0.01	0.11	0.09	0.06	0.00	0.00	0.01
Czech	0.02	0.04	0.07	0.11	0.24	0.37	0.32	0.25	0.00	0.01	0.01	0.00	0.39
Denmark	0.01	0.01	0.10	0.10	0.01	0.08	0.07	0.03	0.01	0.03	0.05	0.02	0.02
Estonia	0.00	0.01	0.21	0.03	0.21	0.00	0.04	0.09	0.00	0.02	0.10	0.18	0.01

Finland	0.01	0.01	0.24	0.05	0.00	0.06	0.07	0.00	0.01	0.03	0.01	0.05	0.04
France	0.00	0.00	0.01	0.08	0.16	0.05	0.01	0.02	0.01	0.03	0.01	0.02	0.05
Germany	0.02	0.05	0.08	0.00	0.02	0.01	0.15	0.12	0.11	0.12	0.05	0.05	0.07
Greece	0.00	0.17	0.13	0.24	0.03	0.00	0.00	0.01	0.02	0.03	0.04	0.06	0.01
Hungary	0.09	0.02	0.01	0.05	0.04	0.05	0.05	0.16	0.14	0.05	0.00	0.02	0.06
Ireland	0.13	0.02	0.02	0.01	0.03	0.02	0.02	0.00	0.05	0.01	0.16	0.18	0.04
Italy	0.08	0.00	0.15	0.03	0.04	0.01	0.01	0.02	0.00	0.02	0.08	0.13	0.00
Latvia	0.00	0.00	0.00	0.01	0.23	0.07	0.06	0.02	0.00	0.02	0.12	0.20	0.17
Lithuania	0.02	0.06	0.00	0.01	0.31	0.08	0.08	0.01	0.00	0.00	0.01	0.11	0.19
Macedonia	0.01	0.26	0.31	0.40	0.20	0.05	0.00	0.03	0.11	0.13	0.14	0.04	0.07
Moldova	0.02	0.04	0.07	0.09	0.34	0.10	0.01	0.04	0.02	0.14	0.10	0.09	0.14
Netherlands	0.01	0.00	0.11	0.12	0.14	0.00	0.01	0.00	0.04	0.09	0.14	0.01	0.02
Norway	0.24	0.10	0.02	0.00	0.10	0.01	0.00	0.05	0.04	0.00	0.02	0.00	0.03
Poland	0.00	0.00	0.01	0.17	0.27	0.08	0.01	0.10	0.03	0.02	0.00	0.03	0.14
Portugal	0.00	0.00	0.00	0.09	0.20	0.28	0.02	0.02	0.00	0.10	0.01	0.03	0.21
Romania	0.03	0.08	0.01	0.10	0.23	0.11	0.04	0.00	0.02	0.00	0.00	0.09	0.16
Russia	0.02	0.03	0.00	0.01	0.05	0.01	0.01	0.00	0.00	0.00	0.02	0.04	0.02
Serbia	0.05	0.18	0.10	0.25	0.13	0.04	0.01	0.05	0.05	0.02	0.00	0.02	0.06
Slovakia	0.00	0.01	0.01	0.08	0.16	0.25	0.11	0.11	0.00	0.01	0.03	0.02	0.22
Slovenia	0.20	0.09	0.06	0.10	0.02	0.01	0.04	0.00	0.00	0.01	0.00	0.01	0.00
Spain	0.01	0.17	0.06	0.23	0.04	0.03	0.00	0.00	0.02	0.11	0.03	0.02	0.02
Sweden	0.03	0.11	0.00	0.01	0.29	0.02	0.01	0.23	0.14	0.06	0.02	0.15	0.12
Switzerland	0.00	0.02	0.07	0.01	0.07	0.05	0.00	0.00	0.03	0.01	0.00	0.00	0.03
Turkey	0.03	0.05	0.00	0.15	0.16	0.09	0.02	0.02	0.15	0.12	0.05	0.02	0.09
Ukraine	0.03	0.01	0.06	0.02	0.15	0.12	0.24	0.03	0.01	0.09	0.13	0.16	0.20
UK	0.02	0.01	0.00	0.02	0.01	0.06	0.16	0.01	0.01	0.00	0.07	0.08	0.05
							1961–1990						
Albania	0.10	0.00	0.00	0.02	0.03	0.16	0.07	0.10	0.02	0.04	0.05	0.03	0.05
Austria	0.03	0.39	0.25	0.14	0.02	0.00	0.01	0.01	0.04	0.01	0.11	0.12	0.00
Belgium	0.00	0.05	0.06	0.00	0.01	0.00	0.02	0.00	0.01	0.00	0.01	0.03	0.01
Bulgaria	0.00	0.12	0.01	0.04	0.05	0.05	0.17	0.03	0.01	0.10	0.08	0.08	0.10
Denmark	0.01	0.09	0.01	0.05	0.00	0.03	0.02	0.03	0.01	0.10	0.09	0.07	0.01
Finland	0.00	0.13	0.05	0.00	0.01	0.26	0.09	0.01	0.12	0.00	0.02	0.02	0.24
France	0.01	0.03	0.05	0.05	0.01	0.00	0.00	0.06	0.08	0.11	0.01	0.16	0.00
Greece	0.01	0.02	0.00	0.07	0.24	0.28	0.24	0.01	0.06	0.21	0.16	0.05	0.30
Hungary	0.04	0.04	0.01	0.03	0.01	0.00	0.00	0.00	0.00	0.00	0.15	0.31	0.00
Ireland	0.01	0.07	0.02	0.04	0.00	0.01	0.00	0.08	0.25	0.14	0.00	0.00	0.00
Italy	0.00	0.00	0.00	0.01	0.11	0.10	0.12	0.02	0.02	0.12	0.01	0.00	0.15
Netherlands	0.15	0.00	0.07	0.11	0.12	0.01	0.00	0.01	0.02	0.03	0.07	0.17	0.02
Norway	0.16	0.00	0.03	0.07	0.03	0.02	0.00	0.00	0.00	0.03	0.07	0.06	0.01
Poland	0.03	0.02	0.13	0.26	0.05	0.00	0.04	0.05	0.05	0.10	0.01	0.04	0.00
Portugal	0.00	0.02	0.00	0.02	0.01	0.05	0.00	0.01	0.00	0.02	0.04	0.15	0.02
Romania	0.03	0.12	0.02	0.09	0.00	0.01	0.10	0.10	0.11	0.00	0.01	0.00	0.01
Spain	0.02	0.02	0.00	0.01	0.01	0.04	0.01	0.01	0.07	0.01	0.03	0.03	0.02
Sweden	0.17	0.10	0.01	0.01	0.00	0.00	0.00	0.01	0.01	0.02	0.11	0.06	0.00
Switzerland	0.00	0.00	0.01	0.01	0.01	0.03	0.00	0.01	0.04	0.03	0.12	0.01	0.02
Turkey	0.02	0.01	0.00	0.02	0.18	0.23	0.14	0.02	0.05	0.04	0.00	0.02	0.25
UK	0.00	0.09	0.27	0.31	0.02	0.09	0.11	0.03	0.00	0.01	0.01	0.00	0.05

Table S15 Coefficients of determination (R^2) between the first-differences of mean monthly and July-August the Standardised Precipitation-Evapotranspiration Index (SPEI3) and maize yields for 1993–2017 and 1961–1990

Country	Apr	May	Jun	Jul	Aug	Sep	Oct	Jul–Aug
	1993–2017							
Albania	0.00	0.00	0.02	0.05	0.05	0.02	0.01	0.07
Austria	0.11	0.27	0.21	0.02	0.00	0.04	0.00	0.00
Belarus	0.10	0.13	0.05	0.03	0.03	0.00	0.01	0.04
Belgium	0.13	0.22	0.01	0.01	0.09	0.03	0.06	0.05
Bosnia	0.09	0.13	0.10	0.00	0.00	0.09	0.06	0.00
Bulgaria	0.05	0.00	0.08	0.20	0.01	0.00	0.00	0.08
Croatia	0.04	0.13	0.24	0.02	0.01	0.04	0.07	0.01
Czech	0.06	0.11	0.23	0.02	0.00	0.15	0.03	0.01
France	0.33	0.28	0.01	0.01	0.01	0.04	0.09	0.01
Germany	0.08	0.19	0.05	0.00	0.00	0.05	0.02	0.00
Greece	0.03	0.03	0.01	0.00	0.02	0.05	0.07	0.00
Hungary	0.01	0.14	0.24	0.08	0.02	0.00	0.00	0.05
Italy	0.34	0.19	0.10	0.02	0.00	0.00	0.00	0.01
Lithuania	0.02	0.00	0.06	0.14	0.02	0.01	0.00	0.07
Macedonia	0.10	0.00	0.08	0.18	0.13	0.11	0.11	0.18
Moldova	0.10	0.19	0.01	0.02	0.04	0.05	0.04	0.04
Netherlands	0.20	0.10	0.17	0.01	0.00	0.03	0.00	0.00
Poland	0.26	0.12	0.01	0.03	0.01	0.00	0.00	0.03
Portugal	0.05	0.01	0.04	0.04	0.10	0.04	0.02	0.09
Romania	0.00	0.04	0.00	0.00	0.01	0.00	0.01	0.01
Russia	0.03	0.04	0.00	0.11	0.01	0.06	0.01	0.04
Serbia	0.13	0.15	0.10	0.00	0.00	0.04	0.03	0.00
Slovakia	0.01	0.16	0.26	0.06	0.00	0.09	0.07	0.01
Slovenia	0.11	0.14	0.00	0.01	0.04	0.04	0.01	0.03
Spain	0.16	0.05	0.06	0.01	0.04	0.05	0.03	0.02
Switzerland	0.03	0.04	0.07	0.03	0.00	0.00	0.05	0.01
Turkey	0.06	0.02	0.01	0.00	0.00	0.00	0.00	0.00
Ukraine	0.11	0.13	0.05	0.01	0.01	0.02	0.05	0.01
	1961–1990							
Albania	0.02	0.11	0.01	0.02	0.06	0.01	0.03	0.04
Austria	0.00	0.05	0.29	0.13	0.05	0.00	0.06	0.10
Belgium	0.00	0.00	0.01	0.00	0.11	0.10	0.12	0.03
Bulgaria	0.00	0.00	0.07	0.05	0.01	0.06	0.09	0.01
France	0.00	0.07	0.01	0.02	0.12	0.00	0.01	0.01
Greece	0.00	0.10	0.00	0.01	0.04	0.11	0.02	0.00
Hungary	0.00	0.00	0.01	0.04	0.07	0.07	0.10	0.05
Italy	0.06	0.12	0.03	0.00	0.14	0.00	0.04	0.01
Netherlands	0.00	0.00	0.00	0.01	0.13	0.02	0.00	0.06
Poland	0.02	0.19	0.04	0.01	0.09	0.25	0.01	0.06
Portugal	0.00	0.03	0.09	0.04	0.02	0.01	0.04	0.06
Romania	0.01	0.22	0.20	0.22	0.06	0.14	0.15	0.14
Spain	0.05	0.01	0.01	0.09	0.23	0.09	0.00	0.14
Switzerland	0.01	0.02	0.00	0.09	0.23	0.21	0.18	0.18
Turkey	0.01	0.01	0.01	0.00	0.00	0.04	0.07	0.00

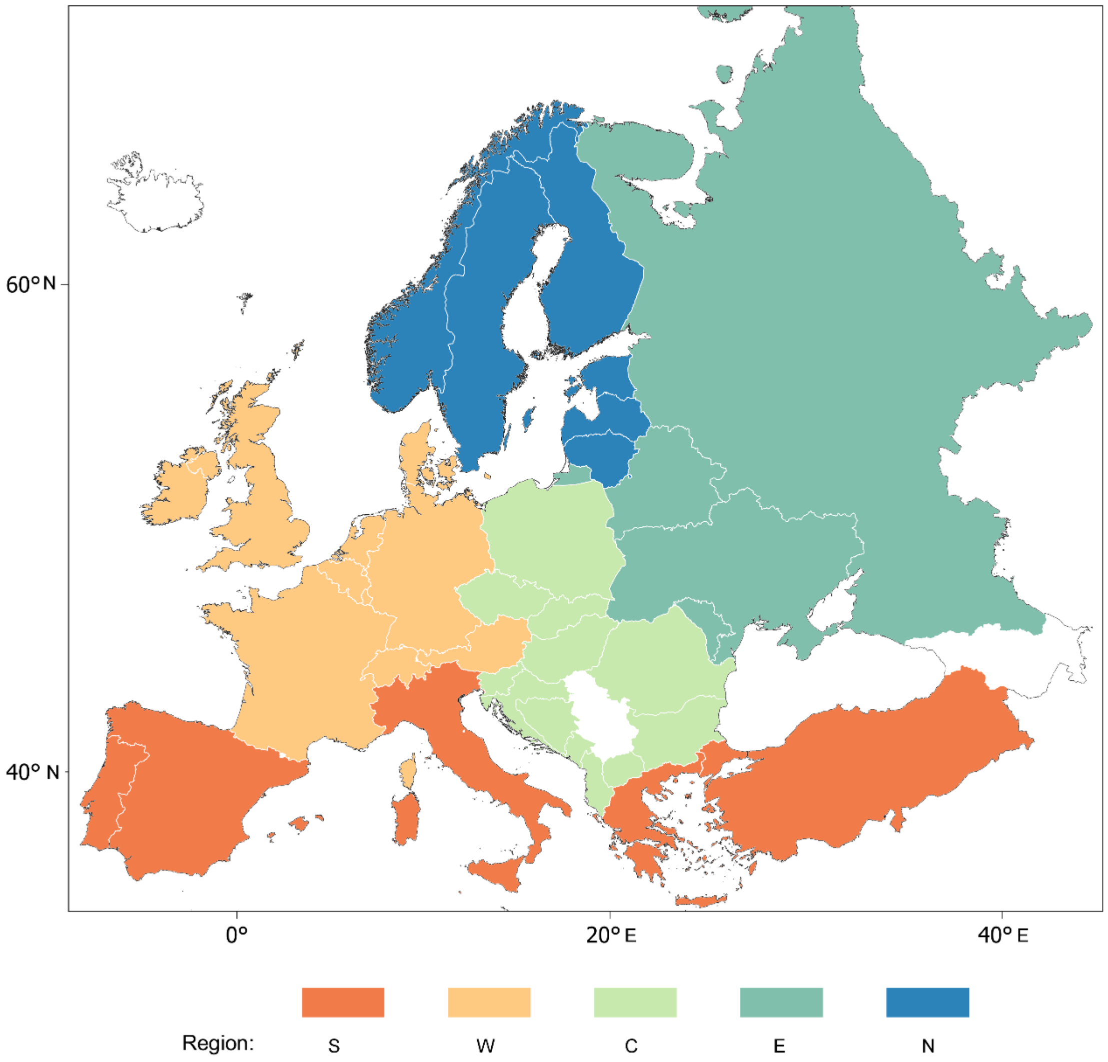

Fig. S3 Countries and regions of the study area. SE = South Eastern Europe, S = Southern Europe, W = Western Europe, C = Central Europe, E = Eastern Europe, N = Northern Europe
